# SNAP23 deficiency triggers Trim21 mitochondrial translocation to suppress TFAM-mediated oxidative metabolism and drive chemoresistance in colorectal cancer

**DOI:** 10.1038/s41419-025-08252-1

**Published:** 2025-11-22

**Authors:** Abudushalamu Yalikun, Bingjie Guan, Jiawei Pan, Haonan Chen, Jingfeng Cai, Ziyan Zhu, Runkai Zhou, Bowen Xie, Youdong Liu, Jikun Li

**Affiliations:** 1 Department of General Surgery, Shanghai General Hospital, Shanghai Jiao Tong University School of Medicine, Shanghai, China; 2 Department of General Surgery, Sir Run Run Shaw Hospital, School of Medicine, Zhejiang University, Hangzhou, Zhejiang China; 3 Department of Gastric Surgery, Fudan University Shanghai Cancer Center, Shanghai, China; 4 Department of Oncology, Shanghai Medical College, Fudan University, Shanghai, China

**Keywords:** Cancer metabolism, Apoptosis

## Abstract

Chemoresistance is a major cause of poor prognosis in colorectal cancer (CRC), and its molecular mechanisms urgently need elucidation. The cell membrane protein SNAP23, known for its role in vesicle secretion, also promotes CRC cell growth. However, its role in tumor chemotherapy remains unclear. This study reveals a novel function of SNAP23, independent of vesicle transport, mediating crosstalk between the cell membrane and mitochondria to influence the chemotherapeutic response to oxaliplatin (OXA). Mechanistically, SNAP23 arrests Trim21, causing its accumulation near the cell membrane and away from mitochondria. This reduces the ubiquitination and degradation of the mitochondrial transcription factor A (TFAM), enhancing mitochondrial oxidative metabolism and increasing oxidative phosphorylation (OXPHOS) and reactive oxygen species (ROS) production, ultimately heightening the sensitivity of cancer cells to OXA. The unique regulatory function of SNAP23 in the chemotherapeutic response of colorectal cancer may provide a potential target for chemotherapy sensitization.

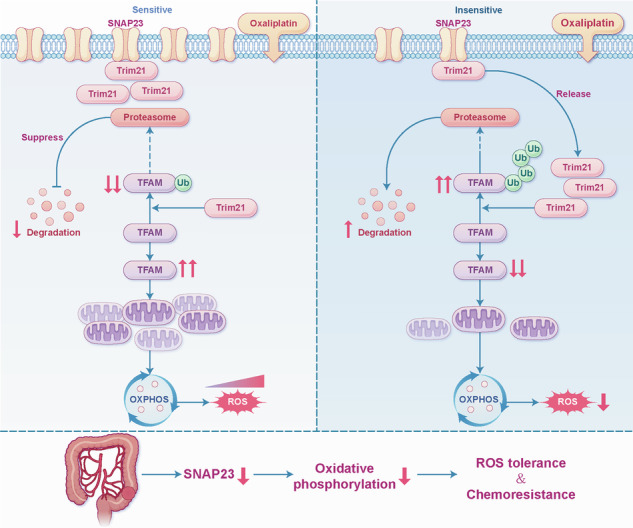

## Introduction

Colorectal cancer (CRC) persists as the second most prevalent cause of cancer-related deaths globally [1]. Chemotherapy remains a cornerstone in the non-surgical management of CRC. Oxaliplatin (OXA), a commonly employed chemotherapeutic drug for advanced-stage CRC, faces challenges due to drug resistance, leading to suboptimal patient outcomes [2]. Therefore, there is a pressing necessity to develop efficacious approaches to augment chemosensitivity, which could significantly enhance the survival rates and prognoses of individuals afflicted with CRC.

Oxidative stress in tumor cells is characterized by the imbalance between the production of reactive oxygen species (ROS) and the cell’s antioxidant defense mechanisms. Tumor cells are exposed to elevated levels of ROS, which, when surpassing a critical threshold, can inflict significant molecular damage, resulting in cell death. This phenomenon is exploited by various anticancer therapies, such as chemotherapy [3–6]. Chemoresistance remains a primary cause of therapeutic failure in cancer treatment. Cancer cells disrupt the redox homeostasis through ROS regulation mechanisms, contributing to tumor progression and chemoresistance [7, 8].

Synaptosomal-associated protein 23 (SNAP23) is an integral component of the SNARE complex, which orchestrates the fusion of intracellular vesicles with membranes to regulate exocytosis [9–11]. SNAP23 exhibits high expression across various cell types and plays a pivotal role in the pathogenesis of multiple cancer forms [12–14]. Our previous studies have corroborated SNAP23’s facilitation of CRC progression [15]. As a potential regulator of cancer cell metabolism, SNAP23 modulates the levels of oxidative phosphorylation (OXPHOS) within mitochondria, thereby influencing the advancement of CRC [16]. Furthermore, ROS, by-products of cellular respiration, serve as critical modulators in intracellular signaling pathways. Hence, we hypothesize that ROS-mediated damage to cancer cells is subject to the regulatory influence of SNAP23 on OXPHOS.

In our investigation, we observed a significant downregulation of SNAP23 expression within tissues exhibiting resistance to OXA in CRC. This downregulation is associated with a parallel decline in the rate of OXPHOS and ROS levels, which contributes to the attenuation of oxidative stress typically induced by OXA. Mechanistically, SNAP23 exerts a competitive inhibition on Trim21-mediated ubiquitination, leading to the subsequent degradation of mitochondrial transcription factor A (TFAM) was suppressed. As a result, diminished SNAP23 levels precipitate an increase in Trim21 release, which captures TFAM and impairs both OXPHOS and ROS production. Our findings provide novel insights into potential biomarkers for predicting therapeutic outcomes and identifying targets for improving chemosensitivity in CRC treatment.

## Results

### Clinical significance of SNAP23 expression in CRC

**Fig. 1:**
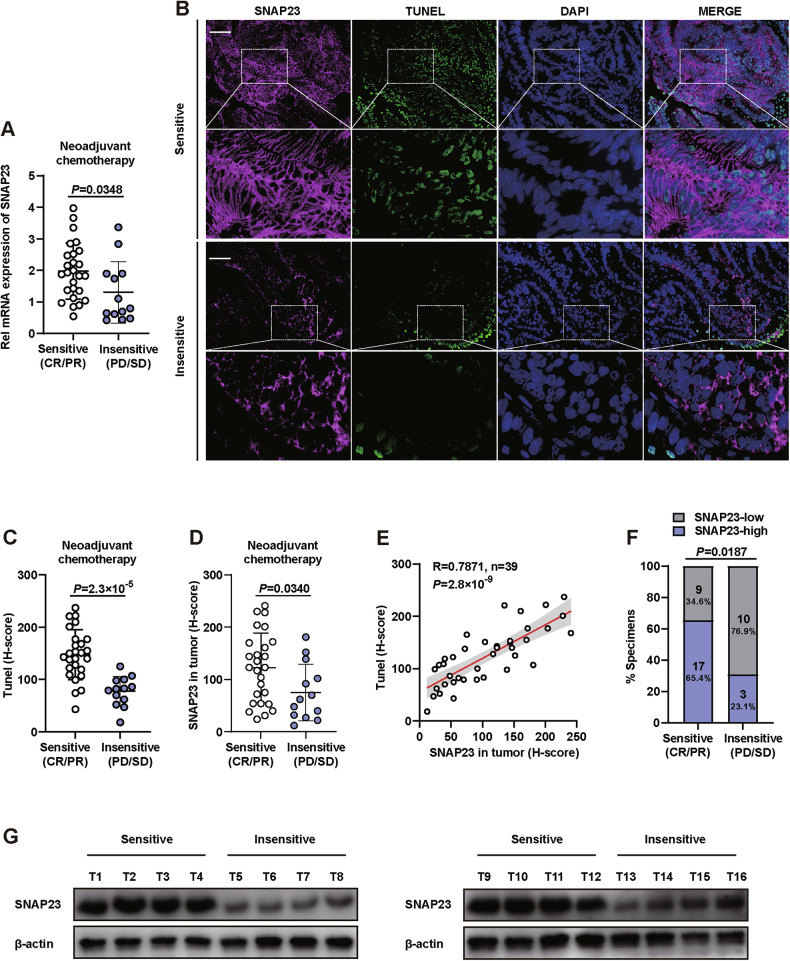
Clinical significance of SNAP23 expression in colorectal cancer (CRC). **A** Quantitative real-time PCR analysis of SNAP23 expression in CRC biopsies (*n* = 39) prior to neoadjuvant chemotherapy (NACT). **B** Representative immunohistochemistry (IHC) images of TUNEL and SNAP23 staining in therapy-sensitive (*n* = 26) and therapy-insensitive (*n* = 13) CRC tissues. Scale bars, 20 μm. **C** Quantification of fluorescence intensity to evaluate the coexpression of SNAP23 and TUNEL. **D** Distribution of SNAP23 expression levels between therapy-insensitive and therapy-sensitive groups. **E** Correlation analysis between IHC scores of SNAP23 and TUNEL levels in CRC patients (*n* = 39) using two-sided Pearson correlation. **F** Comparison of SNAP23 expression levels between therapy-insensitive and therapy-sensitive groups. **G** Western blot analysis of SNAP23 protein levels in therapy-sensitive (*n* = 8) and therapy-insensitive (*n* = 8) CRC tissues. Data are means ± SD. Two-sided Student’s *t* test (**A**, **C**, and **D**). Two-sided Pearson correlation analysis (**E**). Fisher’s exact test (**F**).

We investigated the clinical significance of SNAP23 expression in CRC. Initially, we evaluated whether SNAP23 could serve as a marker for the efficacy of OXA-based neoadjuvant chemotherapy (NACT). To understand the role of SNAP23 in chemoresistance, we observed lower SNAP23 expression in biopsies from therapy-insensitive patients (PD/SD) compared to therapy-sensitive patients (CR/PR) (Fig. 1A). Next, immunohistochemical (IHC) staining combined with terminal deoxynucleotidyl transferase dUTP nick-end labeling (TUNEL) assay demonstrated that low SNAP23 expression significantly reduced tumor cell damage in specimens from insensitive CRC patients (Fig. 1B). The number of SNAP23-positive and TUNEL-positive tumor cells was increased in specimens from sensitive CRC patients, indicating that SNAP23 enhances OXA-induced DNA damage and apoptosis (Fig. 1C, D). Correlation analysis revealed that high SNAP23 expression was significantly associated with increased tumor damage (Fig. 1E). SNAP23 expression correlated with NACT efficacy, with 76.9% of insensitive patients exhibiting low SNAP23 expression, while 65.4% of sensitive patients showed high SNAP23 expression (Fig. 1F).

Finally, we isolated proteins from tumor tissues of therapy-insensitive and therapy-sensitive patients and identified them via immunoblotting for protein markers. This analysis indicated that SNAP23 expression was lower in therapy-insensitive patients (Fig. 1G).

### CRC cells with SNAP23 depletion are tolerate to ROS-inducing chemotherapy

**Fig. 2:**
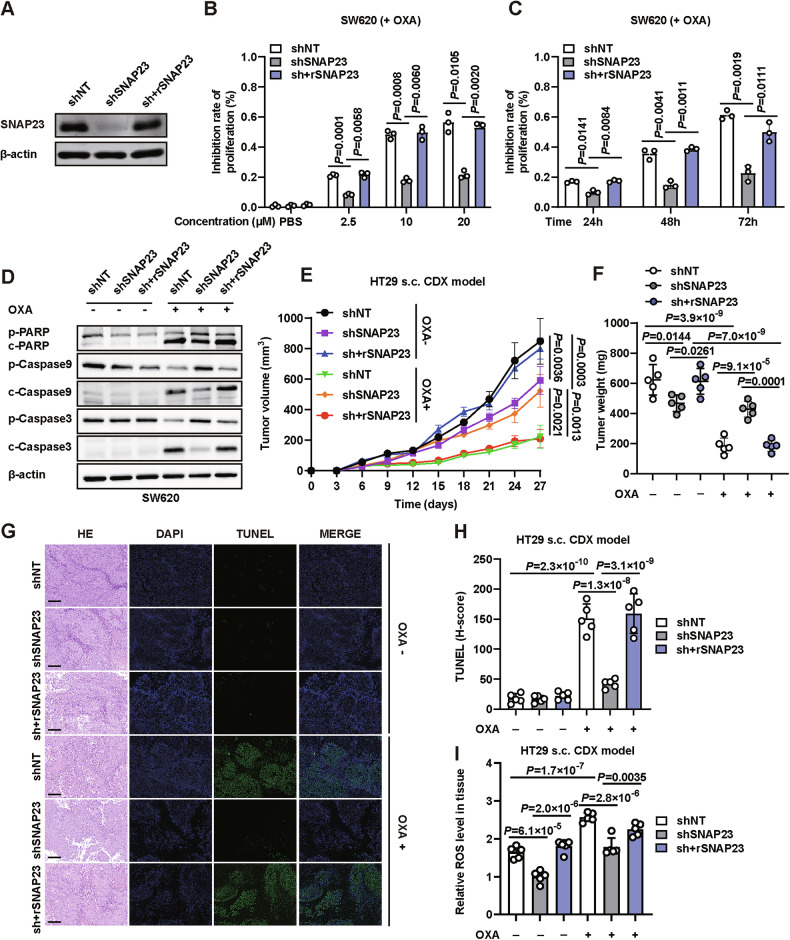
CRC cells with SNAP23 depletion are tolerate to ROS-inducing chemotherapy. **A** Western blot analysis of SNAP23 expression in SW620 cells transfected with shNC, shSNAP23, or sh+rSNAP23 plasmids. **B** Cells were treated with varying concentrations (2.5 µM, 10 µM, 20 µM) of oxaliplatin (OXA) for 72 hours. The inhibition rate was assessed using the SRB assay. **C** Real-time cell analysis using the SRB assay to determine the inhibition rates of OXA (10 µM) over time. **D** Western blot analysis of cleaved PARP, cleaved caspase-9, and cleaved caspase-3 expression in control, SNAP23-knockdown, and SNAP23-restored SW620 cells treated with OXA (10 µM, 72 h). Graphical representation of tumor volumes (**E**) and weights (**F**) in control, SNAP23-knockdown, and SNAP23-restored HT29-induced CDX tumors in nude mice subjected to intraperitoneal injection of OXA (7.5 mg/kg) in the drinking water (*n* = 5). PBS was used as a control. **G** Representative TUNEL and H&E staining images of paraffin-embedded subcutaneous tumor sections. Scale bars, 50 μm. **H** Quantification of fluorescence intensity. **I** ROS levels in HT29-induced CDX tumors. *n* = 5 mice per group. Data are means ± SD. One-way ANOVA with Tukey’s multiple comparisons test (**B**, **C**, **E**, **F**, **H**, **I**).

In our previous study, we identified that SNAP23 regulates cell growth in CRC [16]. Consequently, we focused on elucidating the relationship between SNAP23 and chemoresistance. To knockdown SNAP23 expression, we established SW620 and HT29 cell lines stably expressing shRNAs targeting SNAP23 (Fig. 2A and Supplementary Fig. S1A). To verify the role of SNAP23 in OXA treatment, we treated CRC cells with varying concentrations and durations of OXA. We observed that SNAP23 knockdown significantly enhanced resistance to OXA, notably reducing the cell inhibition rate (Fig. 2B, C, Supplementary Fig. S1B, C).

We further investigated the molecular mechanisms by which SNAP23 inhibition affects cell death pathways during OXA treatment. In terms of apoptosis, we found markedly decreased levels of cleaved caspase-3 in CRC cells treated with OXA following SNAP23 knockdown (Fig. 2D and Supplementary Fig. S1D). Similarly, the upstream activators of caspase-3, PARP and caspase-9, exhibited reduced cleavage (Fig. 2D and Supplementary Fig. S1D).

Given that SNAP23 knockdown significantly enhanced drug sensitivity in vitro, we next assessed the anti-tumor activity in vivo using HT29 cells with stable SNAP23 depletion. We observed reduced tumor weights and volumes in the SNAP23 knockdown group compared to the control group. However, SNAP23 deficiency combined with OXA treatment also exhibited a resistant effect (Fig. 2E–G and Supplementary Fig. S1E). The knockdown of SNAP23 inhibited the tumorigenicity of CRC cells and significantly increased tolerance to OXA-based chemotherapy in nude mice (Fig. 2E–G and Supplementary Fig. S1E). Furthermore, pathological analysis revealed increased apoptosis in the control and SNAP23-restored tumors (Fig. 2H). As expected, the ROS levels were significantly decreased in CRC cells isolated from SNAP23 knockdown xenograft tissues compared to those in the control group and the SNAP23 restored group (Fig. 2I). These results indicate that the resistance of tumor cells to OXA is indeed associated with the expression levels of SNAP23.

### SNAP23 mediates chemoresistance through down-regulating ROS level in tumors

**Fig. 3:**
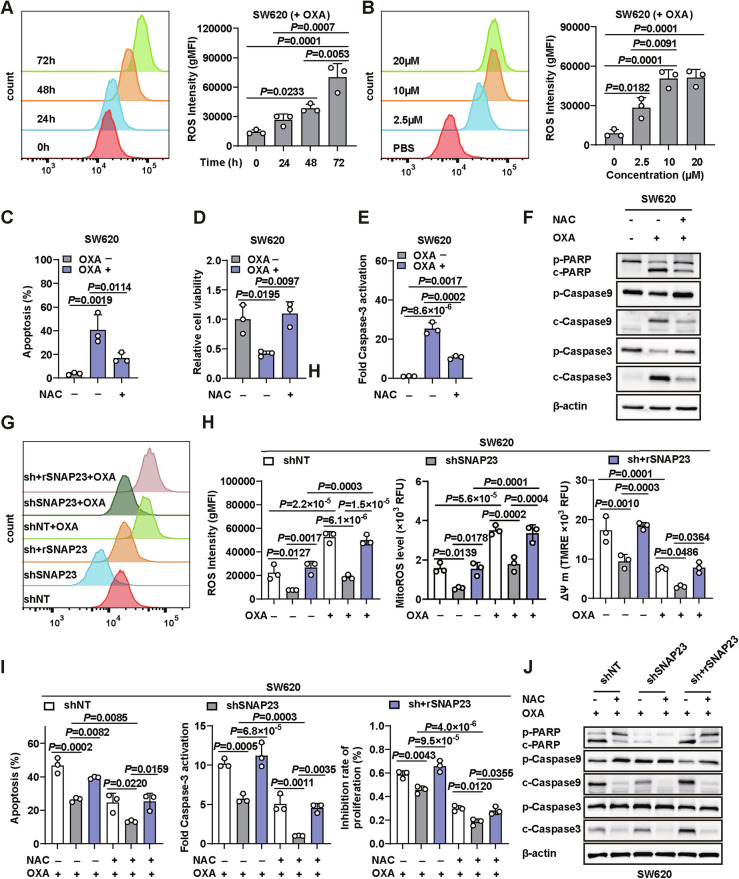
SNAP23 mediates chemoresistance through down-regulating ROS level in tumors. **A** Cells were treated with varying concentrations (2.5 µM, 10 µM, 20 µM) of OXA for 72 h. Intracellular ROS levels were measured using a DCFH-DA probe via flow cytometry (FC) in parental cells. **B** Intracellular ROS levels at different time points were measured using a DCFH-DA probe via FC following treatment with 10 µM OXA in parental cells. **C**–**F** Parental cells were treated with PBS or OXA (10 µM, 72 h) in combination with 5 mM N-acetyl-cysteine (NAC). FC and quantification analysis (**C**) with Annexin V/PI staining were used to evaluate the percentages of live cells (Annexin V−/PI−) and apoptotic cells (Annexin V+/PI+). Cell viability (**D**) of SW620 cells was measured using the SRB assay. Caspase-3 activation (**E**) was measured using an Caspase-3 Activity Assay kit. Western blot analysis (**F**) of cleaved PARP, cleaved caspase-9, and cleaved caspase-3 expression. Representative FC plot (**G**) and statistical results of (**H**) total ROS levels. **I** Changes in mitochondrial ROS levels, mitochondrial membrane potential (ΔΨ m) of control, SNAP23-knockdown, and SNAP23-restored SW620 cells under PBS or OXA treatment (10 µM, 72 h). **I**, **J** Control, SNAP23-knockdown, and SNAP23-restored SW620 cells were treated with OXA (10 µM, 72 h) with or without 5 mM NAC. FC and quantification analysis (**I**) with Annexin V/PI staining were used to evaluate the percentages of live cells (Annexin V−/PI−) and apoptotic cells (Annexin V+/PI+). Cell inhibition rate (**I**) of SW620 cells was measured using the SRB assay. Caspase-3 activation (**I**) was measured using an Caspase-3 Activity Assay kit. Western blot analysis (**J**) of cleaved PARP, cleaved caspase-9, and cleaved caspase-3 expression. Data are means ± SD. One-way ANOVA with Tukey’s multiple comparisons test (**A**–**E**, **H**, **I**).

We evaluated the effects of OXA on ROS levels in our in vitro model and found that OXA treatment at different concentrations and durations induced ROS generation in parental cells (Fig. 3A, B, Supplementary Fig. S2A, B). Initially, parental SW620 and HT29 cells treated with OXA exhibited a significant increase in apoptotic cells. Concurrently, the ROS scavenger N-acetyl-cysteine (NAC) partially rescued the cells from apoptosis (Fig. 3C, D, Supplementary Figs. S2C, D, S3A, S4A). We further investigated the cell death pathway associated with SNAP23 during OXA treatment. For apoptosis, caspase-3 activation was notably increased, as measured by a caspase-3 activity assay kit (Fig. 3E and Supplementary Fig. S2E). Consistent with these findings, we observed markedly increased levels of cleaved PARP, caspase-9, and caspase-3 (Fig. 3F and Supplementary Fig. S2F).

Given the rescue effect observed with NAC treatment, we first measured total ROS and mitochondria ROS (mitoROS) levels and found that SNAP23 deficiency, with or without OXA treatment, significantly decreased total ROS and mitoROS levels compared to both the control and restored groups (Fig. 3G, H and Supplementary Fig. S2G, H). Consistent with the decreased mitochondrial membrane potential (ΔΨ m) (Fig. 3H and Supplementary Fig. S2H). To determine whether OXA-induced apoptosis was mediated via ROS production, we utilized NAC to neutralize the increased ROS. Our results demonstrate that OXA exerts its anti-tumor effects by inducing ROS-dependent apoptosis and proliferation inhibition in tumor cells. Intriguingly, the opposite effect and increased ROS levels were observed in SNAP23-restored cells (Fig. 3H–J and Supplementary Fig. S2H–J). Importantly, NAC markedly rescued the cell death and inhibition induced by OXA in CRC cells (Fig. 3I, J, Supplementary Figs. S2I, J, S3B, S4B). Moreover, NAC treatment suppressed the activation of PARP, caspase-9, and caspase-3 induced by OXA treatment (Fig. 3I, J and Supplementary Fig. S2I, J). In summary, the decreased ROS levels resulting from SNAP23 deficiency, in combination with OXA-based chemotherapy, which inhibits apoptosis, might be critical for drug resistance.

### SNAP23 depletion cells retains better mitochondrial function and integrity with less mitochondrial content and respiratory capacity

Our previous studies identified SNAP23 as a critical regulator of the mitochondrial metabolic phenotype [16]. Additionally, mitochondrial TFAM was found to be decreased in CRC cells with suppressed SNAP23 expression. To elucidate the mechanisms by which ROS-mediated damage to cancer cells is influenced by SNAP23 regulation of OXPHOS in response to OXA, we focused on mitochondrial function in the context of drug resistance, given that mitochondria are the primary source of ROS in cells.

**Fig. 4:**
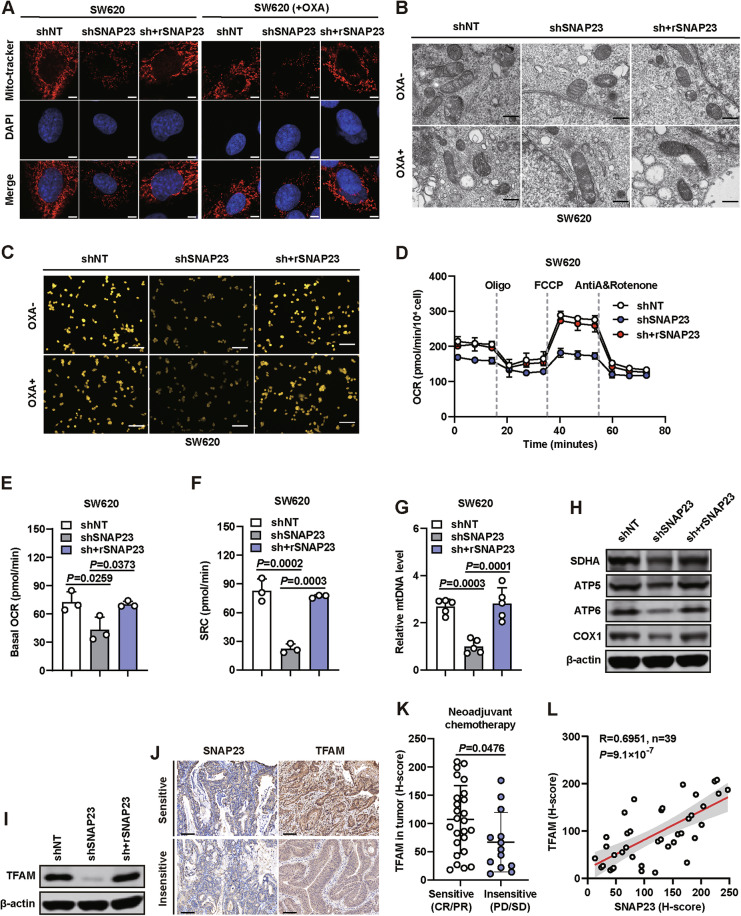
SNAP23 depletion cells retains better mitochondrial function and integrity with less mitochondrial content and respiratory capacity. **A** Confocal images of SW620 cells treated with or without OXA with MitoTracker-labeled mitochondria. Scale bar, 10 μm. **B** Representative transmission electron microscopy (TEM) images showing mitochondrial morphological changes with or without OXA treatment. Scale bar, 500 nm. **C** Representative images of mitochondrial membrane potential assessed by TMRM staining. Scale bar, 50 μm. **D**–**F** Oxygen consumption rate (OCR) analysis (**D**) using Seahorse analysis in control, SNAP23-knockdown, and SNAP23-restored SW620 cells. Basal OCR (**E**) and (**F**) spare respiratory capacity (SRC) were measured in SW620 cells. **G** Relative mtDNA levels in control, SNAP23-knockdown, and SNAP23-restored SW620 cells measured by qPCR. **H** Western blot analysis of mitochondrial protein expression levels. **I** Western blot analysis of TFAM expression levels in control, SNAP23-knockdown, and SNAP23-restored SW620 cells. **J**–**L** Representative immunohistochemistry (IHC) staining images (**J**) and analysis of TFAM (**K**) expression levels in therapy-sensitive (*n* = 26) and therapy-insensitive (*n* = 13) CRC tissues. Scale bars, 50 μm. **L** Correlation analysis of protein levels between SNAP23 and TFAM in CRC tissues. Data are means ± SD. One-way ANOVA with Tukey’s multiple comparisons test (**E**–**G**). Two-sided Student’s *t* test (**K**). Two-sided Pearson correlation analysis (**L**).

We first investigated the subcellular localization and expression of mitochondria in CRC cells. Confocal microscope showed an weaken mitochondrial mass in SW620 and HT29 cells transfected with shSNAP23, suggesting that SNAP23 may contribute to mitochondrial maintenance. Notably, treatment with OXA alone did not significantly alter mitochondrial mass in these cells, indicating that the observed changes were specifically associated with SNAP23 depletion rather than a direct effect of OXA (Fig. 4A and Supplementary Fig. S5A). Transmission electron microscopy (TEM) analysis revealed distinct mitochondrial morphological changes following 72 h of oxaliplatin (OXA) treatment. In both parental and SNAP23-restored cells, OXA exposure induced partial disintegration of mitochondrial cristae, a phenotype that was not observed in untreated controls. In contrast, SNAP23-deficient (shSNAP23) cells maintained relatively intact mitochondrial ultrastructure regardless of OXA treatment (Fig. 4B). These findings suggest that SNAP23 plays a critical role in mediating OXA-induced mitochondrial damage. Mitochondrial membrane potential was significantly reduced in SNAP23-knockdown cells compared to controls, as measured by decreased TMRM fluorescence intensity (Fig. 4C and Supplementary Fig. S5B). OXA treatment caused moderate but significant depolarization in both parental and SNAP23-restored cells, while SNAP23-deficient cells showed no additional response to OXA beyond their baseline depolarized state. These findings indicate SNAP23 is essential for maintaining mitochondrial membrane potential and modulates OXA-induced depolarization.

The metabolic phenotype was assessed using a Seahorse analyzer in SW620 and HT29 cells following SNAP23 knockdown and restoration. Basal oxygen consumption rate (OCR) and spare respiratory capacity (SRC) were significantly lower in SNAP23-deficient cells compared to control and SNAP23-restored groups (Fig. 4D–F and Supplementary Fig. S5C–E). As expected, mtDNA copy number was significantly reduced in SNAP23-deficient cells compared to control and SNAP23-restored cells (Fig. 4G and Supplementary Fig. S5F).

Furthermore, OXPHOS protein expression levels, including COX1, SDHA, ATP5A1, and ATP6V1E1, were significantly downregulated in SNAP23-knockdown cells (Fig. 4H and Supplementary Fig. S5G), but remained at similar levels to control cells in the SNAP23-restored group. We also confirmed that TFAM expression was downregulated in both SNAP23-knockdown HT29 and SW620 cells (Fig. 4I and Supplementary Fig. S5H). IHC staining of 39 CRC tissue samples revealed that TFAM expression was significantly higher in sensitive tissues compared to insensitive tissues (Fig. 4J, K), which correlated with SNAP23 expression (Fig. 4L).

Collectively, these findings suggest that resistant cells (shSNAP23) maintain enhanced mitochondrial efficiency and structural stability despite reduced mitochondrial mass, respiratory capacity and mitochondrial membrane potential. Consequently, these cells exhibit attenuated ROS generation upon OXA treatment, which contributes to their sustained chemoresistance.

### TFAM overcomes chemoresistance through regulating ROS levels in tumors

We further investigated the role of TFAM in the context of SNAP23-attenuated glycolysis and drug resistance in CRC cells. To this end, we induced TFAM overexpression in both SNAP23-silenced cells and SNAP23-stably expressing cells.

**Fig. 5:**
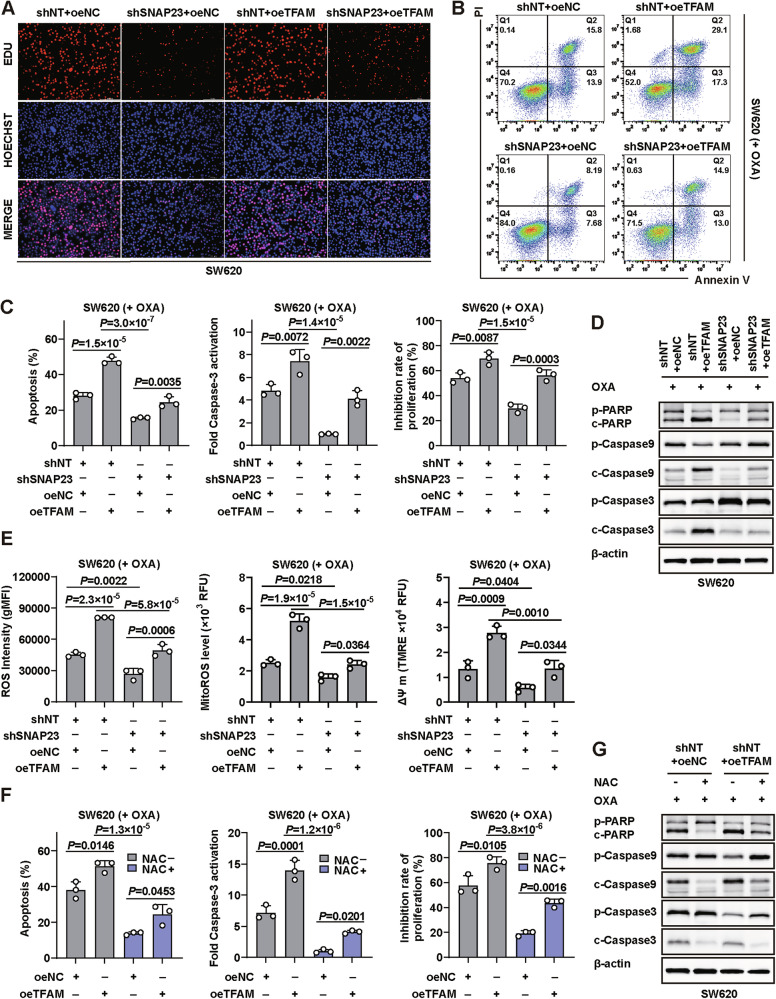
TFAM overcomes drug resistance through regulate ROS levels in tumors. **A** The proliferative abilities of SW620 cells were detected by EdU assays. Scale bar, 100 μm. **B**–**E** The oeNC or oeTFAM plasmids were transfected into SNAP23-silenced cells or SNAP23-stably expressing cells, respectively. These cells were treated with OXA (10 µM, 72 h). Flow cytometry (FC) (**B**) and quantification analysis (**C**) with Annexin V/PI staining were used to evaluate the percentages of live cells and apoptotic cells. Cell inhibition rate (**C**) of SW620 cells was measured using the SRB assay. Caspase-3 activation (**C**) was measured using an Caspase-3 Activity Assay kit. Western blot analysis (**D**) of cleaved PARP, cleaved caspase-9, and cleaved caspase-3 expression. **E** Statistical results of total ROS levels. Changes in mitochondrial ROS levels, mitochondrial membrane potential (ΔΨ m). **F**, **G** TFAM-stably expressing and TFAM-overexpressing SW620 cells were treated with OXA (10 µM, 72 h) with or without 5 mM NAC. FC and quantification analysis (**F**) with Annexin V/PI staining were used to evaluate the percentages of live cells and apoptotic cells. Cell inhibition rate (**F**) of SW620 cells was measured using the SRB assay. Caspase-3 activation (**F**) was measured using an Caspase-3 Activity Assay kit. Western blot analysis (**G**) of cleaved PARP, cleaved caspase-9, and cleaved caspase-3 expression. Data are means ± SD. One-way ANOVA with Tukey’s multiple comparisons test (**C**, **E**, **F**).

Initially, we utilized the EDU assay to examine the impact of TFAM overexpression on cellular proliferation. Our findings indicated that the knockdown of SNAP23 inhibited cell proliferation, consistent with our previous experimental results. However, TFAM overexpression did not affect cell proliferation in either SNAP23-stable expressing cells or SNAP23-knockdown cells (Fig. 5A and Supplementary Fig. S6A). TFAM overexpression in SNAP23-stably expressing cells (shNT+oeTFAM) led to increased cell death and inhibition upon OXA treatment. Notably, TFAM overexpression in SNAP23-silenced cells (shSNAP23+oeTFAM) significantly overcame drug resistance (Fig. 5B, C, Supplementary Fig. S7A, B). Additionally, the levels of cleaved PARP, caspase-9, and caspase-3 were higher in TFAM-overexpressing cells compared to control cells (Fig. 5D and Supplementary Fig. S7C).

To determine whether TFAM overexpression induced apoptosis through the regulation of ROS production, we measured ROS levels in both SNAP23-silenced and SNAP23-stably expressing cells. Our results indicated that ROS generation was elevated in TFAM-overexpressing cells. Furthermore, TFAM overexpression in SNAP23-silenced cells (shSNAP23+oeTFAM) partially restored total ROS and mitoROS levels. Consistent with the increased mitochondrial membrane potential (ΔΨ m) (Fig. 5E and Supplementary Fig. S7D, E).

We then utilized NAC to neutralize the increased ROS in TFAM-overexpressing cells. Tumor cells exhibited a tendency towards drug resistance, as cell death and inhibition were notably rescued by NAC treatment (Fig. 5F, Supplementary Figs. S7F, S8A, B). Moreover, NAC treatment inhibited the activation of PARP, caspase-9, and caspase-3 induced by OXA treatment (Fig. 5F, G and Supplementary Fig. S7F, G).

### TFAM over-expression overcomes OXA resistance in cell-derived xenograft (CDX) models

**Fig. 6:**
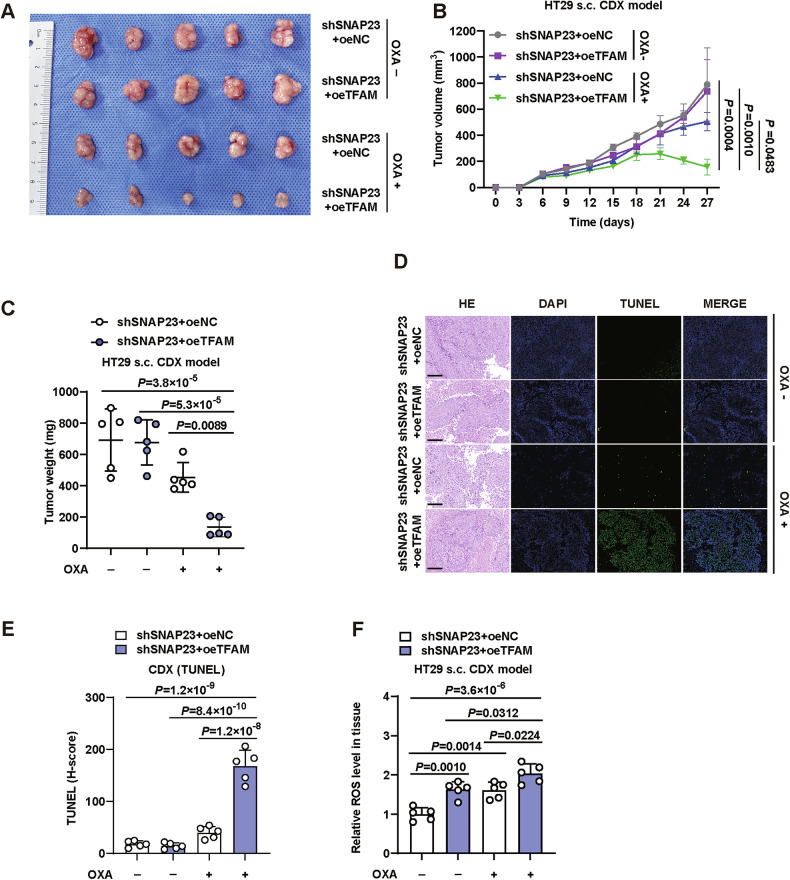
TFAM over-expression overcomes OXA resistance in cell-derived xenograft (CDX) models. **A** Photographs of the excised tumors. Graphical representation of tumor volumes (**B**) and weights (**C**) in control and TFAM-overexpressing HT29-induced CDX tumors in nude mice subjected to intraperitoneal injection of OXA (7.5 mg/kg) in the drinking water (*n* = 5). PBS was used as a control. **D** Representative TUNEL and H&E staining images of paraffin-embedded subcutaneous tumor sections. Scale bars, 50 μm. **E** Quantification of fluorescence intensity. **F** ROS levels in HT29-induced CDX tumors. *n* = 5 mice per group. Data are means ± SD. One-way ANOVA with Tukey’s multiple comparisons test (**B**, **C**, **E**, **F**).

In the final phase, the preclinical evaluation of TFAM over-expression’s impact on OXA resistance was conducted using animal models. For the CDX models, NOD mice were subcutaneously injected with HT29 cells, followed by OXA administration. All mice survived the treatment period, and none exhibited significant body weight loss (>15%) or signs of infection or wounds (Fig. 6A).

Tumors derived from SNAP23 silencing cells were resistant to OXA. However, when TFAM was over-expressed, there was a substantial reduction in tumor mass (Fig. 6B, C). Correspondingly, the shSNAP23+oeTFAM group treated with OXA exhibited significantly higher cell death compared to the other three groups (Fig. 6D, E). Intriguingly, TFAM over-expression group also exhibited an increase in ROS levels compared with paired TFAM stable groups in tumors, strongly suggesting that ROS level of tumor tissues may influence their chemotherapy efficiency through regulated by TFAM (Fig. 6F).

These results indicate that TFAM overexpression enhances the sensitivity of CRC cells to OXA treatment in vivo. This effect appears to be mediated through elevated ROS levels in the tumor tissues, suggesting that ROS regulation by TFAM plays a crucial role in modulating chemotherapy efficacy.

### SNAP23 exerts a competitive inhibition on Trim21-mediated ubiquitination degradation of TFAM

**Fig. 7:**
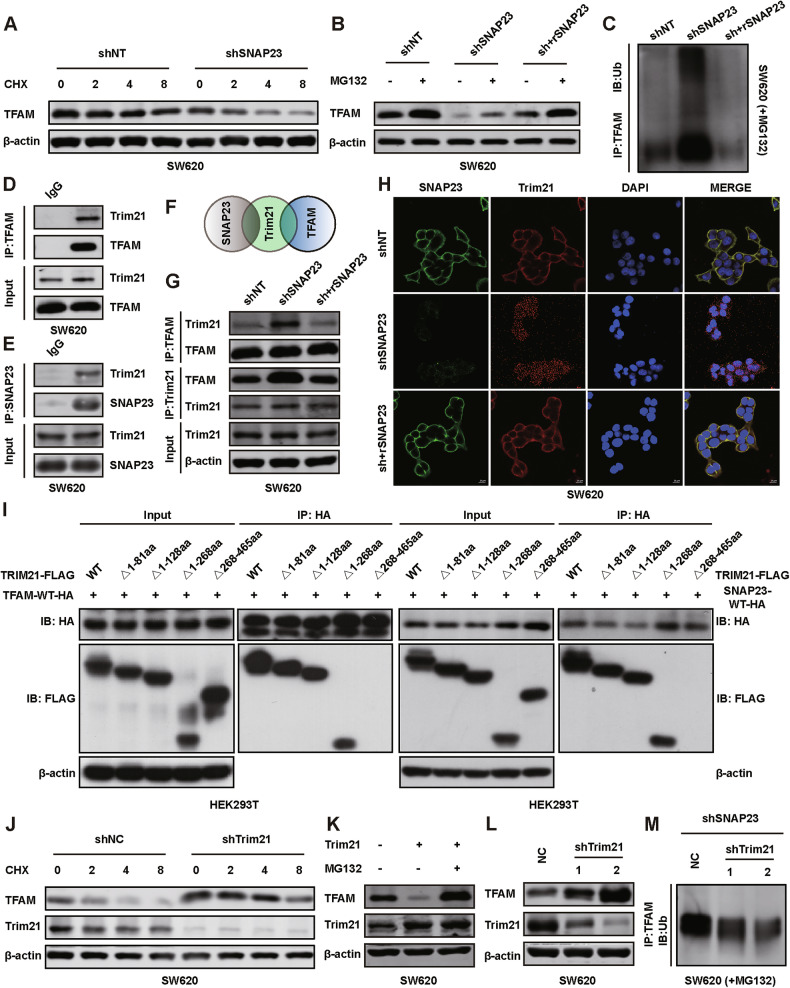
SNAP23 exerts a competitive inhibition on Trim21-mediated ubiquitination degradation of TFAM. **A** Western blot analysis of TFAM expression in control and SNAP23-knockdown SW620 cells after treatment with 100 µg/ml cycloheximide (CHX) for the specified duration. **B** Western blot analysis of TFAM expression in control, SNAP23-knockdown, and SNAP23-restored SW620 cells, treated with or without 10 µM MG132 for 6 h before harvesting. **C** Ubiquitination assays of endogenous TFAM in lysates from control, SNAP23-knockdown, and SNAP23-restored SW620 cells. Cells were treated with 10 µM MG132 for 6 h before harvesting. **D** Interaction of TFAM with Trim21 at the endogenous level. **E** Interaction of SNAP23 with Trim21 at the endogenous level. **F** Mass spectrometry (MS) analysis of SNAP23-associated and TFAM-associated proteins. **G** Western blot analysis of the interaction between TFAM and Trim21 in control, SNAP23-knockdown, and SNAP23-restored SW620 cells. **H** Representative images of IF staining for DAPI (blue), SNAP23 (green) and Trim21 (magenta) in SW620 cells. **I** Co-IP analysis of cell lysates from HEK293T cells expressing the respective deletion mutants. **J** Western blot analysis of TFAM and Trim21 expression in SW620 cells after treatment with 100 µg/ml CHX and transfection with shTrim21. **K** Western blot analysis of TFAM and Trim21 expression in SW620 cells transfected with the indicated plasmids and treated with 10 µM MG132 for 6 h before harvesting. **L** Western blot analysis of TFAM expression in SW620 cells transfected with shTrim21 plasmids. **M** Ubiquitination assays of endogenous TFAM in lysates from SNAP23-knockdown SW620 cells transfected with shTrim21 plasmids and treated with 10 µM MG132 for 6 h before harvesting.

To investigate whether TFAM expression is regulated by SNAP23 through ubiquitin-mediated degradation, we measured the half-life of TFAM in multiple CRC cell lines. We observed that the half-life of TFAM was shortened following the knockdown of endogenous SNAP23 (Fig. 7A and Supplementary Fig. S9A). Concurrently, we observed that pretreatment with the proteasome inhibitor MG132 reduced TFAM degradation in SW620 and HT29 cells(Fig. 7B and Supplementary Fig. S9B). Furthermore, our Co-immunoprecipitation (Co-IP) assay demonstrated that knockdown of SNAP23 promoted TFAM ubiquitination, which was captured by MG132 pretreatment but blocked upon SNAP23 restoration (Fig. 7C and Supplementary Fig. S9C).

We hypothesized that SNAP23 regulates TFAM activity through direct physical interaction. However, initial assessments showed no interaction between SNAP23 and TFAM. To systematically identify potential binding partners of TFAM, we performed mass spectrometry (MS)-based proteomic analysis of the immunoprecipitated fractions of TFAM and SNAP23 in SW620 cells. Surprisingly, the E3 ubiquitin ligase Trim21 co-immunoprecipitated with both SNAP23 and TFAM (Supplementary Fig. S9D). Subsequent Co-IP assays confirmed the endogenous interactions of Trim21 with SNAP23 and TFAM in SW620 and HT29 cells (Fig. 7D–F, Supplementary Fig. S9E, F).

To further explore the localization and expression levels of the endogenous interaction between SNAP23 and Trim21 in CRC cells, we knocked down SNAP23 and then restored its expression. Co-IP results indicated that SNAP23 and TFAM competitively bind to Trim21 (Fig. 7G and Supplementary Fig. S9G). After staining SNAP23 and Trim21, confocal microscope showed that SNAP23 and Trim21 interacted at the cell membrane, and upon knockdown of SNAP23, a greater amount of Trim21 is released into the cytoplasm (Fig. 7H and Supplementary Fig. S9H). In addition, Trim21 has been previously identified as an E3 ubiquitin ligase involved in regulating protein degradation [17, 18]. To investigate the structural determinants of the interaction between Trim21 and TFAM, a series of Trim21 deletion mutants, each lacking specific domains, was generated. Co-IP analysis revealed that the Trim21 mutants removing amino acids 1–81aa, 1–128aa and 1-268aa retained the ability to bind TFAM, whereas the Trim21 mutant removing amino acids 268–465aa lost this capacity (Fig. 7I). These results indicate that the region spanning amino acids 268–465aa, which includes the PRY/SPRY domain of Trim21, is crucial for its interaction with TFAM. Besides, these Trim21 deletion mutants also retained the ability to bind SNAP23 except the Trim21 mutant removing amino acids 268–465aa (Fig. 7I). These results indicate that the PRY/SPRY domain, ranging from 268aa to 465aa of Trim21, was responsible for its interaction with SNAP23 (Fig. 7I). It further verified that SNAP23 and TFAM competitively bind to Trim21.

Consistent with these findings, the half-life of TFAM was prolonged following the knockdown of endogenous Trim21 (Fig. 7J and Supplementary Fig. S9I), suggesting that Trim21 primarily controls TFAM expression through post-translational modifications regulated by SNAP23.

We observed that ectopic overexpression of Trim21 significantly downregulated TFAM abundance in SW620 and HT29 cells. However, this Trim21-mediated downregulation of TFAM was blocked by MG132 pretreatment (Fig. 7K and Supplementary Fig. S9J). Consistently, TFAM expression was upregulated following Trim21 knockdown via shRNA treatment in multiple cell lines (Fig. 7L and Supplementary Fig. S8K). Additionally, our results demonstrated that Trim21 promotes TFAM ubiquitination (Fig. 7M and Supplementary Fig. S9L). Collectively, these findings indicate that SNAP23 competitively binds Trim21, thereby modulating TFAM abundance through ubiquitin-mediated degradation.

### SNAP23 mediates the redistribution of Trim21, facilitating TFAM ubiquitination and degradation

**Fig. 8:**
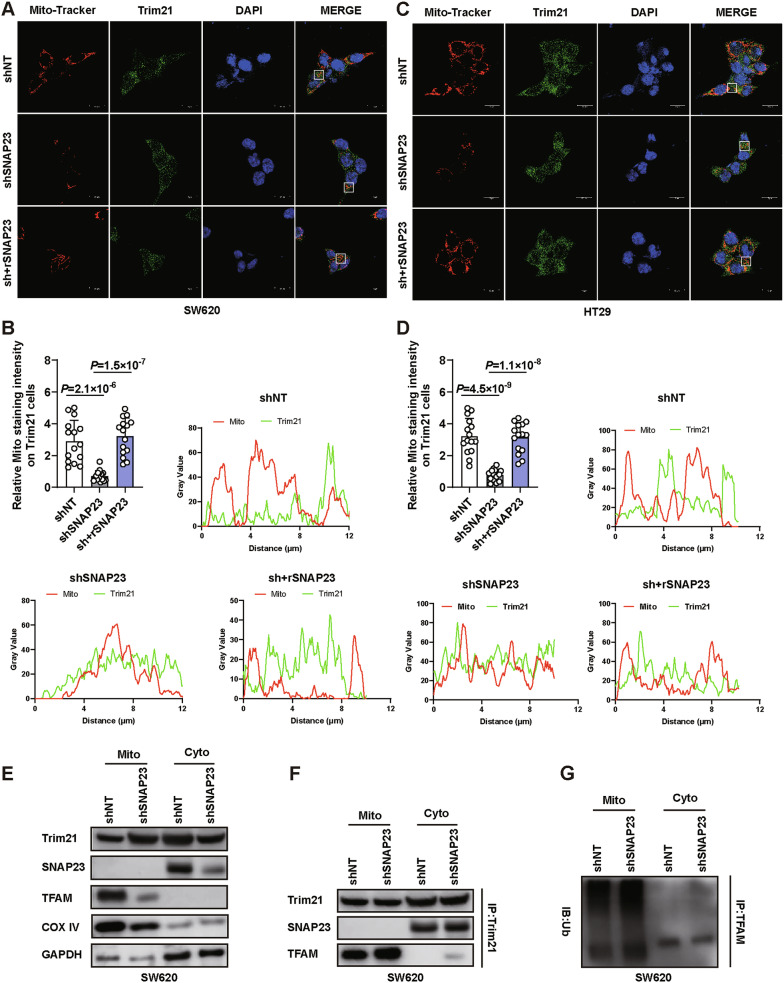
SNAP23 facilitates the translocation of Trim21 into the mitochondria. Representative images (**A**), statistical analysis of the Mito/Trim21 ratio (**B**) and colocalization analysis (**B**) of Trim21 and mitochondria in SW620 cells. Scale bar, 15 μm. Representative images (**C**), statistical analysis of the Mito/Trim21 ratio (**D**) and colocalization analysis (**D**) of Trim21 and mitochondria in SW620 cells. Scale bar, 15 μm. **E** Western blot analysis of COX IV, GAPDH, SNAP23, Trim21 and TFAM expression in mitochondrial and cytoplasmic fractions isolated from control (shNT) and SNAP23-knockdown (shSNAP23) SW620 cells. **F** Subcellular fractionation followed by Co-IP was performed in control (shNT) and SNAP23-knockdown (shSNAP23) SW620 cells. Mitochondrial and cytoplasmic fractions were isolated, and was immunoprecipitated from each fraction. Western blot were probed for SNAP23, Trim21, and TFAM. **G** Subcellular fractionation followed by Co-IP was performed in control (shNT) and SNAP23-knockdown (shSNAP23) SW620 cells treated with MG132. Mitochondrial and cytoplasmic fractions were isolated, and was immunoprecipitated from each fraction. Western blot were probed for Ubiquitin. Data are means ± SD. One-way ANOVA with Tukey’s multiple comparisons test (**B**, **D**).

To investigate the subcellular distribution of Trim21 and its potential association with mitochondria, we performed immunofluorescence staining using anti-Trim21 antibodies in combination with MitoTracker staining in both SW620 and HT29 colorectal cancer cell lines (Fig. 8A, B). Under basal conditions, Trim21 displayed diffuse cytoplasmic localization with minimal mitochondrial overlap. In contrast, SNAP23 depletion significantly increased Trim21 recruitment to mitochondria, as evidenced by its colocalization with the mitochondria (Fig. 8C, D).

To further validate the endogenous binding sites of Trim21 with SNAP23 and TFAM, we purified and extracted mitochondria from cells. Our results indicated that Trim21 can be found in both the mitochondria and cytoplasm (Fig. 8E). Mitochondria and cytoplasm were purified from cells pretreated with MG132. Co-IP revealed that Trim21 interacts with SNAP23 in the cytoplasm and with TFAM in the mitochondria (Fig. 8F). Furthermore, the Co-IP assays demonstrated that Trim21 directly promotes TFAM ubiquitination specifically within mitochondria (Fig. 8G). This finding supports our hypothesis that SNAP23 facilitates the translocation of Trim21 into the mitochondria, where it binds to TFAM.

## Discussion

Despite the significance of OXA in the treatment of CRC, resistance to chemotherapy remains a formidable challenge, impeding the overall improvement of therapeutic outcomes [19–22]. The exploration of molecular mechanisms underlying resistance to OXA is essential for overcoming the therapeutic bottleneck in CRC management [23, 24]. In our study, we first determined that a marked reduction in SNAP23 expression in biopsies from therapy-insensitive patients (PD/SD) as compared to therapy-sensitive patients (CR/PR). IHC staining coupled with TUNEL assay and WB detecting analysis confirmed reduced SNAP23 expression in therapy-insensitive patients, suggesting the clinical importance of our findings.

SNAP23 emerges as a pivotal protein, orchestrating the specificity of vesicle docking and fusion [10, 13, 25, 26]. This protein collaborates with synaptic vesicle-associated membrane protein and syntaxin to form a complex that serves as a critical docking site within the general membrane fusion machinery [27]. Recent investigations have increasingly focused on the role of SNAP23 in tumorigenesis and tumor progression [28, 29]. Our previous research has also demonstrated that SNAP23 can modulate the release of TNFα-rich extracellular vesicles and, through LAMB3-dependent mechanisms, facilitate the malignant progression of CRC cells [15].

ROS play a dual role in cancer progression and chemoresistance. Chemotherapeutic agents often elevate intracellular ROS levels, leading to oxidative stress and subsequent cancer cell death [30, 31]. However, cancer cells can modulate or adapt to these elevated ROS levels by upregulating antioxidant defense mechanisms, which can mitigate the cytotoxic effects of ROS and contribute to chemoresistance. To sustain resistance to OXA and further promote tumor progression, cancer cells employ strategies to mitigate excessive ROS production, thereby acquiring resistance to oxidative stress-induced cell death [32]. In this study, we confirmed that OXA treatment induces ROS generation in both CRC cell lines. Unexpectedly, we observed reduced ROS levels in SNAP23 knockdown cells following OXA treatment, which contributes to the persistence of chemoresistance. Understanding the balance between ROS generation and elimination is crucial for developing effective therapeutic strategies to overcome chemoresistance.

To understand more adequately the role of SNAP23 in regulating mitochondrial metabolic processes. We determine the metabolic phenotype by the Seahorse analyzer. The basal oxygen OCR and SRC were decreased in SNAP23 knockdown cells. In our previous study, we also tested that downregulated SNAP23 could suppress glycolysis in CRC cells [16]. To further test this, we determined OXPHOS protein expression levels that a reduction in ATP5A1, knows as ATP synthasome, complex II SDHA and complex IV COX1 from CRC cells transfected shSNAP23. Moreover, the weaken mitochondrial biogenesis revealed in SNAP23 knockdown cells.These findings highlight the multifaceted biological functions of SNAP23 beyond its role in vesicle transport, implicating it in metabolic processes and tumor progression, warranting further investigation.

Mitochondria serve as the primary site for ROS production and redox homeostasis [33, 34]. Consequently, a reduction in mitochondrial biogenesis is likely to result in decreased ROS production, thereby reducing cellular stress and enhancing adaptation to OXA treatment. Notably, studies have revealed that CRC cells transitioning from a rapid growth phase to a slow growth phase can evade chemotherapy, because chemotherapy agents primarily target rapidly dividing cells [35]. Our findings demonstrate that knockdown of SNAP23 significantly inhibits tumor cell proliferation. Intriguingly, the expression of TFAM was also observed to fluctuate concomitantly with changes in SNAP23 levels. However, the expression level of TFAM did not influence the proliferation rate of tumor cells. This finding suggests a potential link between SNAP23 expression and chemoresistance. Our earlier studies reveal that a decrease in SNAP23 levels weakens the OXPHOS of CRC cells [16].

TFAM is an essential nuclear-encoded protein that is translocated to the mitochondria, where it plays a pivotal role in the maintenance, expression, and delivery of mitochondrial DNA (mtDNA) [36, 37]. TFAM is integral to the regulation of mtDNA replication and transcription processes [38]. It is also implicated in the formation of mtDNA nucleomimetic structures, mtDNA repair mechanisms, and the overall stability of mtDNA [39]. Post-translational modifications such as acetylation and phosphorylation modulate TFAM’s binding affinity to mtDNA, thereby influencing mtDNA dynamics [40]. Additionally, TFAM is instrumental in regulating intramitochondrial calcium levels, which are crucial for the activation of mitochondrial transcription [39]. As a master regulator of mitochondrial respiratory chain biogenesis, TFAM enhances mitochondrial OXPHOS, underscoring its significance in preserving mitochondrial function and its potential impact on cellular energy production [41, 42]. Dysregulation of TFAM has been linked to mitochondrial dysfunction and is associated with a spectrum of diseases [43–46].

Our study demonstrates that overexpression of TFAM elevates mitochondrial ROS (mtROS) production and triggers tumor cell apoptosis, a process linked to mitochondrial hyperpolarization which a finding experimentally validated in our work. TFAM, a critical regulator of mtDNA stability and copy number, binds mtDNA to maintain its structural integrity and promote mitochondrial gene transcription [47]. However, when overexpressed, TFAM leads to an uncontrolled increase in mtDNA content, disrupting the stoichiometric balance of electron transport chain (ETC) complexes. This imbalance results in electron leakage and excessive superoxide generation, a primary source of mtROS [48]. Notably, our data confirm that TFAM overexpression induces mitochondrial hyperpolarization (elevated ΔΨm), a phenomenon that further exacerbates ROS accumulation. Hyperpolarization reduces proton leakage across the inner mitochondrial membrane, prolonging electron retention in the ETC and increasing the likelihood of electron escape to oxygen, thereby amplifying mtROS production [49–51]. Colorectal cancer cells increase Ca2+ uptake, which activates phosphodiesterase 2 (PDE2) and inhibits the activity of mitochondrial protein kinase A (PKA), leading to the stabilization of TFAM accumulation in mitochondria [52]. Similarly, Liu et al. found that increased mitochondrial Ca2+ uptake upregulates TFAM expression, promoting mitochondrial biogenesis and increasing mitochondrial ROS production [53]. The in vitro and in vivo models showed that genetic blockade of SNAP23 attenuate the anti-tumor effect of OXA. But overexpression of TFAM could markedly rescue the resistant effect through upregulated the ROS levels. The downregulation of TFAM further clarifies the reason that the inhibition of it eliminates the effect of enhanced OXPHOS.

To further elucidate the mechanism by which SNAP23 regulates TFAM, we utilized mass spectrometry, Co-IP, immunofluorescence and mitochondrial purification assays. These comprehensive analyses confirmed that SNAP23 and Trim21 interacted at the cell membrane, and upon knockdown of SNAP23, a greater amount of Trim21 is released into the cytoplasm. Besides, SNAP23 competitively inhibits Trim21-mediated ubiquitination and subsequent degradation of TFAM. Therefore, we propose that the levels of OXPHOS within tumors are positively regulated via the SNAP23-Trim21-TFAM axis.

The interaction between SNAP23, a membrane-associated protein, and TFAM, a mitochondrial protein, presents a fascinating mechanism that may involve the translocation of the protein across the membrane and subsequent alterations in cellular localization. The factors driving the reduction of SNAP23 levels in the resistant cells of CRC to OXA warrant further elucidation. Additionally, the potential role of SNAP23 in metabolic processes and chemoresistance in other types of tumors is an intriguing subject for future research.

## Methods

### Cell culture

Human embryonic kidney cell line 293T (CL-0005) and human colorectal cancer cell lines HT29 (CL-0118) and SW620 (CL-0225B) were obtained from Procell (Wuhan, China). The cells were cultured in Dulbecco’s Modified Eagle Medium (DMEM) supplemented with 10% fetal bovine serum (FBS) and 1% penicillin-streptomycin at 37 °C in a 5% CO2 atmosphere. Cells were seeded in 6-well plates overnight and transfected with various shRNAs and plasmids using Lipofectamine 3000, following the manufacturer’s protocol. After 8 h, the medium was replaced with fresh DMEM. Cells were subsequently harvested for further analysis as described in the results section. All cell lines were authenticated by short tandem repeat (STR) profiling and routinely tested negative for mycoplasma contamination.

### Clinical samples

Cancer samples were procured from Shanghai General Hospital (Shanghai Jiao Tong University School of Medicine) for this study. CRC samples were collected from patients who had undergone neoadjuvant chemotherapy prior to surgery. Additionally, biopsies of cancer samples were collected before neoadjuvant chemotherapy. The therapeutic response was classified as sensitive (complete response [CR] or partial response [PR]) or insensitive (progressive disease [PD] or stable disease [SD]) to neoadjuvant chemotherapy, independently evaluated by three radiologists blinded to the clinical data (Table 1). This study was approved by the Ethics Committee of Shanghai General Hospital (2020SQ150).

**Table 1:** Clinical characteristics in patients underwent neoadjuvant chemotherapy.

Characteristics	No. of patients	Outcome of chemotherapy		*P*
		Sensitive	Insensitive	
Age				
<65	23	15	9	0.7281
≥65	16	11	4	
Gender				
Male	14	10	4	0.7334
Female	25	16	9	
BMI	39	23.5 (22.5–24.5)	24.0 (23.0–25.0)	0.2267
cT stage				
II	8	6	2	0.3921
III	18	10	8	
IV	13	10	3	
cN stage				
I	8	5	3	0.7903
II	18	13	5	
III	13	8	5	
Differentiation				0.4451
Well-moderate	10	8	2	
Poor	29	18	11	

### Establishment of stable cell lines

Colorectal cancer (CRC) cells were transfected with plasmid vectors using Lipofectamine 3000 (Thermo Fisher Scientific), followed by the selection of positively transfected cells for two weeks until colony formation. The SNAP23 knockdown (shSNAP23), SNAP23 restored/overexpression (rSNAP23), Trim21 knockdown (shTrim21), and TFAM overexpression lentiviruses were obtained from Shanghai Qihe Company, along with non-targeting shRNA controls (shNT/shNC/oeNC). The designed target sequences are listed in Table 2. Transfection efficiency was validated by immunoblotting.

**Table 2:** Key resources used in this study.

Reagent or resource	Source	Identifier
Antibodies		
Rabbit polyclonal anti-SNAP23 (for WB, IP, IHC, IF)	Proteintech	Cat# 10825-1-AP, RRID:AB_2192022
TRIM21 (D1O1D) Rabbit mAb (for WB, IP)	CST	Cat# 92043,RRID:AB_2800177
TFAM (D5C8) Rabbit mAb (for WB, IP, IHC)	CST	Cat# 8076, RRID:AB_10949110
Ubiquitin (E6K4Y) XP® Rabbit mAb (for WB)	CST	Cat# 20326, RRID:AB_3064918
Cleaved PARP (Asp214) (D64E10) XP® Rabbit mAb (for WB)	CST	Cat# 5625, RRID:AB_10699459
Cleaved Caspase-3 (Asp175) (5A1E) Rabbit (for WB)	CST	Cat# 9664, RRID:AB_2070042
Cleaved Caspase-9 (Asp330) (D2D4) Rabbit (for WB)	CST	Cat# 7237, RRID:AB_10895832
COX1 Rabbit mAb (for WB)	Abclonal	Cat# A23123, RRID:AB_3096107
ATP5A1 Rabbit mAb (for WB)	Abclonal	Cat# A11217, RRID:AB_2861524
ATP6V1E1 Rabbit pAb (for WB)	Abclonal	Cat# A3756, RRID:AB_2765253
SDHA Rabbit mAb (for WB)	Abclonal	Cat# A13852, RRID:AB_2861697
HA Tag Recombinant antibody (for IP, WB, CoIP)	Proteintech	Cat# 81290-1-RR,RRID:AB_2935602
DYKDDDDK tag Monoclonal antibody (Binds to FLAG® tag epitope) (for IP, WB, CoIP)	Proteintech	Cat# 66008-4-Ig, RRID:AB_2918475
β-Actin mAb (for WB)	Proteintech	Cat# 66009-1-Ig, RRID:AB_2687938
Goat anti-Mouse IgG (H + L) Secondary Antibody, HRP	ThermoFisher	Cat# 31430; RRID: AB_228307
Goat anti-Rabbit IgG (H + L) Secondary Antibody, HRP	ThermoFisher	Cat# 31460; RRID: AB_228341
IRDye® 680RD Goat anti-Mouse IgG (H + L) Secondary Antibody	LI-COR Biosciences	Cat# 925-68070, RRID:AB_2651128
IRDye® 680RD Goat anti-Rabbit IgG (H + L) Secondary Antibody	LI-COR Biosciences	Cat# 925-68071, RRID:AB_2721181
IRDye® 800CW Goat anti-Mouse IgG (H + L) Secondary Antibody	LI-COR Biosciences	Cat# 925-32210, RRID:AB_2687825
IRDye® 800CW Goat anti-Rabbit IgG (H + L) Secondary Antibody	LI-COR Biosciences	Cat# 925-32211, RRID:AB_2651127
Biological samples		
Colorectal cancer biopsies sample	Shanghai General Hospital	N/A
Tumor tissues of CRC patients	Shanghai General Hospital	N/A
Chemicals		
DMEM Medium	Gibco	Cat# 61965059
Trypsin-EDTA (0.5%)	Gibco	Cat# 15400054
Opti-MEM I Reduced Serum Medium	Gibco	Cat# 31985047
Oxaliplatin	MCE	Cat# 61825-94-3
N-Acetyl-L-cysteine (NAC)	MCE	Cat# 616-91-1
Dimethyl sulfoxide	MCE	Cat# 67-68-5
Cycloheximide	MCE	Cat# 66-81-9
MG132	MCE	Cat# 133407-82-6
TRIzol	ThermoFisher	Cat# 15596026
puromycin	Sigma-Aldrich	Cat# 58-58-2
G418	Sigma-Aldrich	Cat# 108321-42-2
Penicilin/Streptomicin	NCM Biotech	Cat# C100C5
RIPA Buffer	NCM Biotech	Cat# WB3100
Universal Antibody Diluent	NCM Biotech	Cat# WB500D
DAPI	Sigma-Aldrich	Cat# D9542-1
Critical commercial assays		
TUNEL BrightGreen Apoptosis Detection Kit	Vazyme	Cat# A112-03
Cell Counting Kit-8	DOJINDO	Cat# CK04-100
CellTiter-Glo® 2.0 Cell Viability Assay	Promega	Cat# G9242
FITC Annexin V Apoptosis Detection Kit with PI	BioLegend	Cat# 640914
Caspase-3 Activity Assay Kit	Abcam	Cat# ab252897
Reactive Oxygen Species Assay Kit	Beyotime	Cat# S0033S
Immunoprecipitation Kit with Protein A + G Agarose Gel	Beyotime	Cat# P2197M
Seahorse XFp Cell Mito Stress Test Kit	Agilent Technologies	Cat# 103015-100
Oligonucleotides		
shSNAP23	This paper	GCAGTATCCTGGGAAATCT
SNAP23 (rSNAP23)	This paper	GAAGCATCTGGTAACCT
shTrim21-1	This paper	GGAAGTGGAAATTGCAATAAA
shTrim21-2	This paper	CTGCTGCAGGAGGTGATAATT
TRIM21		**F :**5’-CTCGGATCCGCCACCATGGCTTCAGCAGCACGCTT-3’ **R :**5’-CCCTCTAGACTCGAGATAGTCAGTGGATCCTTGTG-3
TRIM21 (△1–81aa)	This paper	**F :**5’-CTCGGATCCGCCACCATGGAGGCCAGAGAGGGCAC-3’ **R :**5’-CCCTCTAGACTCGAGATAGTCAGTGGATCCTTGTG-3
TRIM21 (△1–128aa)	This paper	**F :**5’-CTCGGATCCGCCACCATGGAGGAGGCTGCACAGGA-3’ **R :**5’-CCCTCTAGACTCGAGATAGTCAGTGGATCCTTGTG-3
TRIM21 (△1–268aa)	This paper	**F :**5’-CTCGGATCCGCCACCATGCTCAGGAGTGTGTGCCA-3’ **R :**5’-CCCTCTAGACTCGAGATAGTCAGTGGATCCTTGTG-3
TRIM21 (△268–465aa)	This paper	**F1 :**5’-CTCGGATCCGCCACCATGGCTTCAGCAGCACGCTT-3’ **R1 :**5’-TCCAATATTTTCTGGAGAGGTAATATCCAG-3 **F2 :**TATTACCTCTCCAGAAAATATTGGAT **R2 :**5’-CCCTCTAGACTCGAGATAGTCAGTGGATCCTT-3
TFAM	This paper	**F :**5’-CTCGGATCCGCCACCATGGCGTTTCTCCGAAGCAT-3’ **R :**5’-CCCTCTAGACTCGAGACACTCCTCAGCACCATATT-3
Software and algorithms		
GraphPad PRISM 9	GraphPad	https://www.graphpad.com/
ImageJ software	GitHub	https://imagej.net/software/imagej/
FlowJo	BD Bioscience	https://www.bdbiosciences.com/zh-cn/products/software/flowjo-v10-software
Image Studio™ Software	LI-COR Biosciences	https://www.licor.com/bio/image-studio/resources

### Cell inhibition and viability analysis

Three thousand cells per well were seeded in 96-well plates and allowed to grow for the indicated durations. At the experimental endpoint, cells were incubated with medium premixed with 10% CCK8 (Dojindo, Kumamoto, Japan) at 37 °C for 2 h, followed by absorbance measurement at 450 nm. Alternatively, SRB assays were used to determine cell proliferation: cells were fixed with 10% trichloroacetic acid overnight at 4 °C and stained with 0.4% SRB for 20 min at room temperature. After washing with 1% acetic acid and solubilizing the residual dye with 10 mM Tris base solution for 15 min, absorbance was measured at 510 nm. Cell viability assays were performed using the CellTiter-Glo 2.0 Assay Kit (Promega, Madison, WI, USA) and normalized to cellular protein content measured in parallel. Absorbance and chemiluminescence intensity were quantified using a Varioskan Multimode Microplate Reader (Thermo Fisher Scientific). The EdU assay (RiboBio, Guangzhou, China) was also performed to detect the proliferation of CRC cells. Experiments were independently repeated three times.

### Cell apoptosis analysis

Cell apoptosis induced by oxaliplatin was detected using Annexin V/PI (Biolegend, #641904, USA) via flow cytometry. Caspase-3 activity was assessed using the Caspase-3 Activity Assay Kit (Abcam, Cambridge, UK) on a Varioskan Multimode Microplate Reader (Thermo Fisher Scientific) after 6 h. Immunoblotting was performed to determine the levels of cleaved Caspase-3, cleaved Caspase-9, and cleaved PARP.

### TUNEL assay

The TUNEL assay was conducted using a TUNEL Bright Green Apoptosis Detection Kit (Vazyme, Nanjing, China). Paraffin-embedded sections were deparaffinized, rehydrated, permeabilized, and blocked with 3% H₂O₂ for 20 min to inhibit endogenous peroxidase activity. Sections were incubated with TdT reaction cocktails for 60 min at 37 °C, followed by treatment with a streptavidin-HRP reaction mixture for 30 min at 37 °C. Observations were made using a confocal laser scanning microscope (TCS SP5, Leica).

### IHC staining

IHC assays were performed using standard methods as previously reported [54]. Paraffin sections were prepared from colon cancer tissues and mouse subcutaneous tumor tissues. Sections were deparaffinized and rehydrated, and endogenous peroxidase activity was blocked with 3% H₂O₂ for 10 min. Antigen retrieval was performed using sodium citrate buffer or EDTA in a pressure cooker for 10 min at sub-boiling temperature. Samples were blocked with 10% FBS for 1 h at room temperature to prevent nonspecific binding, followed by overnight incubation with primary antibodies at 4 °C. HRP-conjugated secondary antibodies (Dako Cytomation, USA) were applied at room temperature for 30 min. IHC staining scores were calculated as described in our previous report.

### ROS assay

Intracellular ROS levels were measured using a DCFH-DA probe (Beyotime, China) via flow cytometry (BD Biosciences). Data in FCS format were analyzed using FlowJo v10.0.7 (BD Biosciences). Concurrently, mitochondrial ROS levels were quantified using the Cell Meter Fluorimetric Mitochondrial Superoxide Activity Assay Kit, designed for optimal performance with a microplate reader (AAT Bioquest, Sunnyvale, CA, USA), following the manufacturer’s protocol. ROS levels in colon tissue were detected using the Tissue Reactive Oxygen Species Detection Kit (BestBio, Shanghai, China).

### Immunoblotting (IB) and Co-IP

Cell and tissue lysates were extracted using RIPA lysis buffer containing a protease and phosphatase inhibitor cocktail (NCM Biotech, Suzhou, China). For Co-IP analysis, the appropriate antibody was added to the supernatant and incubated at 4 °C overnight. The mixture was then incubated with Pierce Protein A + G Agarose Gel (P2197M, Beyotime) for 1 h at room temperature. Bound proteins were extracted after centrifugation and elution. To assess ubiquitination levels, cells were treated with MG132 (HY-13259, MCE) for 6 h before lysis, followed by Co-IP assay. Equivalent amounts of protein were electrophoresed on SDS-PAGE gels and transferred to PVDF membranes (Millipore, Billerica, MA). The antibodies used for IB and Co-IP assays are listed in Table 2.

### Protein half-life analysis

HT29 and SW620 cells were transfected with the desired constructs. Forty hours post-transfection, cells were incubated with cycloheximide (HY-12320, MCE). Cells were harvested at various time points and analyzed by IB for protein abundance. Mitochondrial function assays.

### Mitochondrial activity assay

Mitochondrial activity was assessed using a Seahorse XF Analyzer (Seahorse Bioscience, Billerica, MA, USA) [16, 42]. Each well in the assay plates was confirmed to be sub-confluent with comparable cell density on the day of the assay, and results were normalized to cell number. Cells seeded in an XF96 plate were sequentially treated with oligomycin (1 μM), FCCP (0.5 μM), and rotenone/antimycin A (0.5 μM each) [42]. Mitochondrial morphological changes during oxaliplatin treatment were observed using transmission electron microscopy (TEM). Absorbance and fluorescence intensity were quantified using a Varioskan Multimode Microplate Reader (Thermo Fisher Scientific). Mitochondrial membrane potential was assessed using the TMRE Mitochondrial Membrane Potential Assay Kit (BioVision, Milpitas, CA, USA) and TMRM (HY-D0984, MCE) imaged via microscopy.

### Mitochondrial purification

Cells were collected by centrifugation at 370 × *g* for 10 min and washed twice with NKM buffer. After resuspension in homogenization buffer, the cells were homogenized on ice using a glass homogenizer until ~60% lysis was achieved. The homogenate was mixed with 2 M sucrose solution and centrifuged at 1200 × *g* for 5 min to remove debris. The supernatant was then centrifuged at 7000 × *g* for 10 min to pellet mitochondria, which were subsequently resuspended in mitochondrial suspension buffer and re-pelleted at 9500 × *g* for 5 min. For complete mitochondrial protein extraction, the pellet was resuspended in protein gel loading buffer and subjected to WB detection.

### Immunofluorescence (IF) microscopy

Fluorescence microscopy was utilized to investigate the subcellular localization and expression of mitochondria in CRC cells. Initially, the cells were incubated with Mito-tracker™ Red CMXRos (Invitrogen™, USA) for 30 min. Subsequently, nuclear staining was performed using DAPI. Finally, the samples were analyzed using a confocal microscope (Leica TCS SP8, Germany). To examine the localization and expression of SNAP23 and Trim21, the cells were fixed with 4% paraformaldehyde for 15 min, followed by permeabilization with 0.2% Triton X-100 for 5 min. The cells were then incubated with primary antibodies specific to SNAP23 and Trim21, and subsequently with a goat anti-rabbit secondary antibody. The samples were imaged using a confocal microscope (Leica TCS SP8, Germany).

### CDX models

We established cell-derived subcutaneous xenograft (CDX) models to evaluate the combined effects of SNAP23 depletion and oxaliplatin treatment. Six-week-old BALB/c nude mice were used for the animal studies. All mice were subcutaneously injected in the armpit with a 5 ×10^6^ single cell suspension and maintained in a specific-pathogen-free (SPF) environment.

Upon successful establishment of the colon cancer xenograft model, the mice received subcutaneous injections of oxaliplatin (7.5 mg/kg) intraperitoneally every three days for a total of five doses [55]. Tumor volumes were measured using a vernier caliper [24] every three days for a total of seven measurements to generate tumor growth curves.

Nude mice were subcutaneously inoculated with HT29 cells transfected with shNT, shSNAP23, sh+rSNAP23, shSNAP23+oeNC, or shSNAP23+oeTFAM, followed by subcutaneous injections of either PBS or oxaliplatin (*n* = 5 mice/group). All animal experiments were conducted in accordance with the guidelines of the Animal Care and Use Committee of Shanghai General Hospital, Shanghai Jiao Tong University School of Medicine.

### Statistical analysis

Data are presented as mean ± standard deviation. An independent sample *t*-test was used for comparisons between two groups. For multiple group comparisons, a one-way ANOVA followed by Tukey’s or Games-Howell post-hoc test (when variances were not homogeneous) was employed. Two-sided Pearson correlation analysis was used to evaluate correlations between variables. The chi-squared test or Fisher’s exact test was used for comparisons of categorical data. A *p*-value < 0.05 was considered statistically significant.

## Supplementary information

Supplementary figure legends

Supplementary Figure 1

Supplementary Figure 2

Supplementary Figure 3

Supplementary Figure 4

Supplementary Figure 5

Supplementary Figure 6

Supplementary Figure 7

Supplementary Figure 8

Supplementary Figure 9

Original Western Blots

Supporting data

## Data Availability

All data generated or analyzed during this study are included in this published article [and its supplementary information files].
