# Supplementary material for: SNAP23 deficiency triggers Trim21 mitochondrial translocation to suppress TFAM-mediated oxidative metabolism and drive chemoresistance in colorectal cancer
Source: Cell Death Dis. 2025 Nov 22;17(1):52. doi: 10.1038/s41419-025-08252-1 (PMC12811313; doi:10.1038/s41419-025-08252-1)
Supplement: Supplementary file 1 — Supplementary figure legends [file 41419_2025_8252_MOESM1_ESM.docx]

# Supplementary Figure 1. CRC cells with SNAP23 depletion are tolerate to ROS-inducing chemotherapy

(A) Western blot analysis of SNAP23 expression in HT29 cells transfected with shNC, shSNAP23, or sh+rSNAP23 plasmids.

(B) Cells were treated with varying concentrations (2.5 µM, 10 µM, 20 µM) of OXA for 72 hours. The inhibition rate was assessed using the SRB assay.

(C) Real-time cell analysis using the SRB assay to determine the inhibition rates of OXA (10 µM) over time.

(D) Western blot analysis of cleaved PARP, cleaved caspase-9, and cleaved caspase-3 expression in control, SNAP23-knockdown, and SNAP23-restored HT29 cells treated with OXA (10 µM, 72 hours).

(E) Photographs of the excised tumors.

Data are means ± SD. One-way ANOVA with Tukey’s multiple comparisons test (B and C).

# Supplementary Figure 2. SNAP23 mediates chemoresistance through down-regulating ROS level in tumors

(A) Cells were treated with varying concentrations (2.5 µM, 10 µM, 20 µM) of OXA for 72 hours. Intracellular ROS levels were measured using a DCFH-DA probe via FC in parental cells.

(B) Intracellular ROS levels at different time points were measured using a DCFH-DA probe via FC following treatment with 10 µM OXA in parental cells.

(C-F) Parental HT29 cells were treated with PBS or OXA (10 µM, 72 hours) in combination with 5 mM NAC. Quantification analysis (C) with Annexin V/PI staining were used to evaluate the percentages of live cells (Annexin V−/PI−) and apoptotic cells (Annexin V+/PI+). Cell viability (D) of HT29 cells was measured using the SRB assay. Caspase-3 activation (E) was measured using an Caspase-3 Activity Assay kit. Western blot analysis (F) of cleaved PARP, cleaved caspase-9, and cleaved caspase-3 expression.

(G-H) Representative FC plots (G) and total ROS (H) analysis of control, SNAP23-knockdown, and SNAP23-restored HT29 cells under PBS or OXA treatment (10 µM, 72 hours). (H) Changes in mitochondrial ROS levels, mitochondrial membrane potential (ΔΨ m).

(I-J) Control, SNAP23-knockdown, and SNAP23-restored HT29 cells were treated with OXA (10 µM, 72 hours) with or without 5 mM NAC. Quantification analysis (I) with Annexin V/PI staining were used to evaluate the percentages of live cells (Annexin V−/PI−) and apoptotic cells (Annexin V+/PI+). Cell inhibition rate (I) of HT29 cells was measured using the SRB assay. Caspase-3 activation (I) was measured using an Caspase-3 Activity Assay kit. Western blot analysis (J) of cleaved PARP, cleaved caspase-9, and cleaved caspase-3 expression.

Data are means ± SD. One-way ANOVA with Tukey’s multiple comparisons test (A, B, C, D, E, H and I).

# Supplementary Figure 3. SNAP23 mediates chemoresistance through down-regulating ROS level in tumors

(A) Parental SW620 cells were treated with PBS or OXA (10 µM, 72 hours) in combination with 5 mM N-acetyl-cysteine (NAC). FC plots with Annexin V/PI staining were used to evaluate the percentages of live cells (Annexin V−/PI−) and apoptotic cells (Annexin V+/PI+).

(B) Control, SNAP23-knockdown, and SNAP23-restored SW620 cells were treated with OXA (10 µM, 72 hours) with or without 5 mM NAC. FC plots with Annexin V/PI staining were used to evaluate the percentages of live cells (Annexin V−/PI−) and apoptotic cells (Annexin V+/PI+).

# Supplementary Figure 4. SNAP23 mediates chemoresistance through down-regulating ROS level in tumors

(A) Parental HT29 cells were treated with PBS or OXA (10 µM, 72 hours) in combination with 5 mM N-acetyl-cysteine (NAC). FC plots with Annexin V/PI staining were used to evaluate the percentages of live cells (Annexin V−/PI−) and apoptotic cells (Annexin V+/PI+).

(B) Control, SNAP23-knockdown, and SNAP23-restored HT29 cells were treated with OXA (10 µM, 72 hours) with or without 5 mM NAC. FC plots with Annexin V/PI staining were used to evaluate the percentages of live cells (Annexin V−/PI−) and apoptotic cells (Annexin V+/PI+).

# Supplementary Figure 5. SNAP23 depletion cells retains better mitochondrial function and integrity with less mitochondrial content and respiratory capacity

(A) Confocal images of HT29 cells treated with or without OXA with MitoTracker-labeled mitochondria. Scale bar, 10 μm.

(B) Representative images of mitochondrial membrane potential assessed by TMRM staining. Scale bar, 50 μm.

(C-E) Oxygen consumption rate (OCR) analysis (C) using Seahorse analysis in control, SNAP23-knockdown, and SNAP23-restored HT29 cells. Basal OCR (D) and (E) spare respiratory capacity (SRC) were measured in SW620 cells.

(F) Relative mtDNA levels in control, SNAP23-knockdown, and SNAP23-restored HT29 cells measured by qPCR.

(G) Western blot analysis of mitochondrial protein expression levels.

(H) Western blot analysis of TFAM expression levels in control, SNAP23-knockdown, and SNAP23-restored SW620 cells.

Data are means ± SD. One-way ANOVA with Tukey’s multiple comparisons test (D, E and F).

# Supplementary Figure 6. TFAM overcomes drug resistance through regulate ROS levels in tumors

(A) The proliferative abilities of HT29 cells were detected by EdU assays. Scale bar, 100 μm.

# Supplementary Figure 7. TFAM overcomes chemoresistance through regulating ROS levels in tumors

(A-E) The oeNC or oeTFAM plasmids were transfected into SNAP23-knockdown cells or SNAP23-stably expressing cells, respectively. These cells were treated with OXA (10 µM, 72 hours). Representative FC plots (A) and quantification analysis (B) with Annexin V/PI staining were used to evaluate the percentages of live cells and apoptotic cells. Cell inhibition rate (B) of HT29 cells was measured using the SRB assay. Caspase-3 activation (B) was measured using an Caspase-3 Activity Assay kit. Western blot analysis (C) of cleaved PARP, cleaved caspase-9, and cleaved caspase-3 expression. Representative FC plots (D) and statistical results of (E) total ROS levels. (E) Changes in mitochondrial ROS levels, mitochondrial membrane potential (ΔΨ m).

(F-G) TFAM-stably expressing and TFAM-overexpressing HT29 cells were treated with OXA (10 µM, 72 hours) with or without 5 mM NAC. Quantification analysis (F) with Annexin V/PI staining were used to evaluate the percentages of live cells and apoptotic cells. Cell inhibition rate (F) of HT29 cells was measured using the SRB assay. Caspase-3 activation (F) was measured using an Caspase-3 Activity Assay kit. Western blot analysis (G) of cleaved PARP, cleaved caspase-9, and cleaved caspase-3 expression.

Data are means ± SD. One-way ANOVA with Tukey’s multiple comparisons test (B, E and F).

# Supplementary Figure 8. TFAM overcomes chemoresistance through regulating ROS levels in tumors

(A-B) The oeNC or oeTFAM plasmids were transfected into SNAP23-stably expressing SW620 and HT29 cells, respectively. These cells were treated by OXA (10 µM, 72 hours) with or without 5 mM NAC. Representative FC plots with Annexin V/PI staining were used to evaluate the percentages of live SW620 (A) and HT29 (B) cells and apoptotic cells.

# Supplementary Figure 9. SNAP23 exerts a competitive inhibition on Trim21-mediated ubiquitination degradation of TFAM

(A) Western blot analysis of TFAM expression in control and SNAP23-knockdown HT29 cells after treatment with 100 µg/ml CHX for the specified duration.

(B) Western blot analysis of TFAM expression in control, SNAP23-knockdown, and SNAP23-restored HT29 cells, treated with or without 10 µM MG132 for 6 hours before harvesting.

(C) Ubiquitination assays of endogenous TFAM in lysates from control, SNAP23-knockdown, and SNAP23-restored HT29 cells. Cells were treated with 10 µM MG132 for 6 hours before harvesting.

(D) Mass spectrometry (MS) analysis of SNAP23-associated and TFAM-associated proteins.

(E) Interaction of TFAM with Trim21 at the endogenous level.

(F) Interaction of SNAP23 with Trim21 at the endogenous level.

(G) Western blot analysis of the interaction between TFAM and Trim21 in control, SNAP23-knockdown, and SNAP23-restored HT29 cells.

(H) Representative images of IF staining for DAPI (blue), SNAP23 (green) and Trim21 (magenta) in HT29 cells.

(I) Western blot analysis of TFAM and Trim21 expression in HT29 cells after treatment with 100 µg/ml CHX and transfection with shTrim21.

(J) Western blot analysis of TFAM and Trim21 expression in HT29 cells transfected with the indicated plasmids and treated with 10 µM MG132 for 6 hours before harvesting.

(K) Western blot analysis of TFAM expression in HT29 cells transfected with shTrim21 plasmids.

(L) Ubiquitination assays of endogenous TFAM in lysates from SNAP23-knockdown HT29 cells transfected with shTrim21 plasmids and treated with 10 µM MG132 for 6 hours before harvesting.
