# Supplementary figures and images for: SNAP23 deficiency triggers Trim21 mitochondrial translocation to suppress TFAM-mediated oxidative metabolism and drive chemoresistance in colorectal cancer
Source: Cell Death Dis. 2025 Nov 22;17(1):52. doi: 10.1038/s41419-025-08252-1 (PMC12811313; doi:10.1038/s41419-025-08252-1)

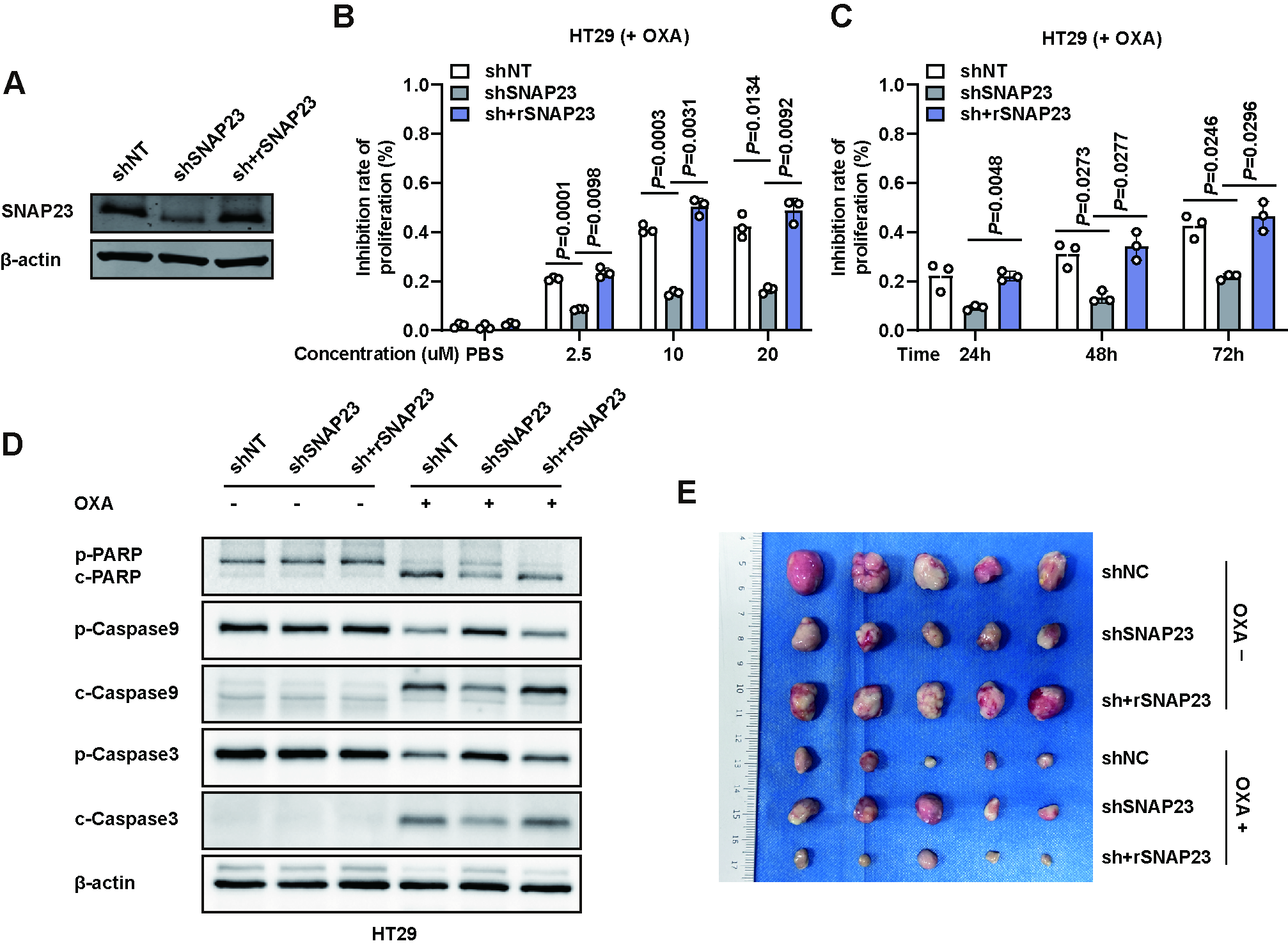

Supplement: Supplementary file 2 — Supplementary Figure 1 [file 41419_2025_8252_MOESM2_ESM.tif]

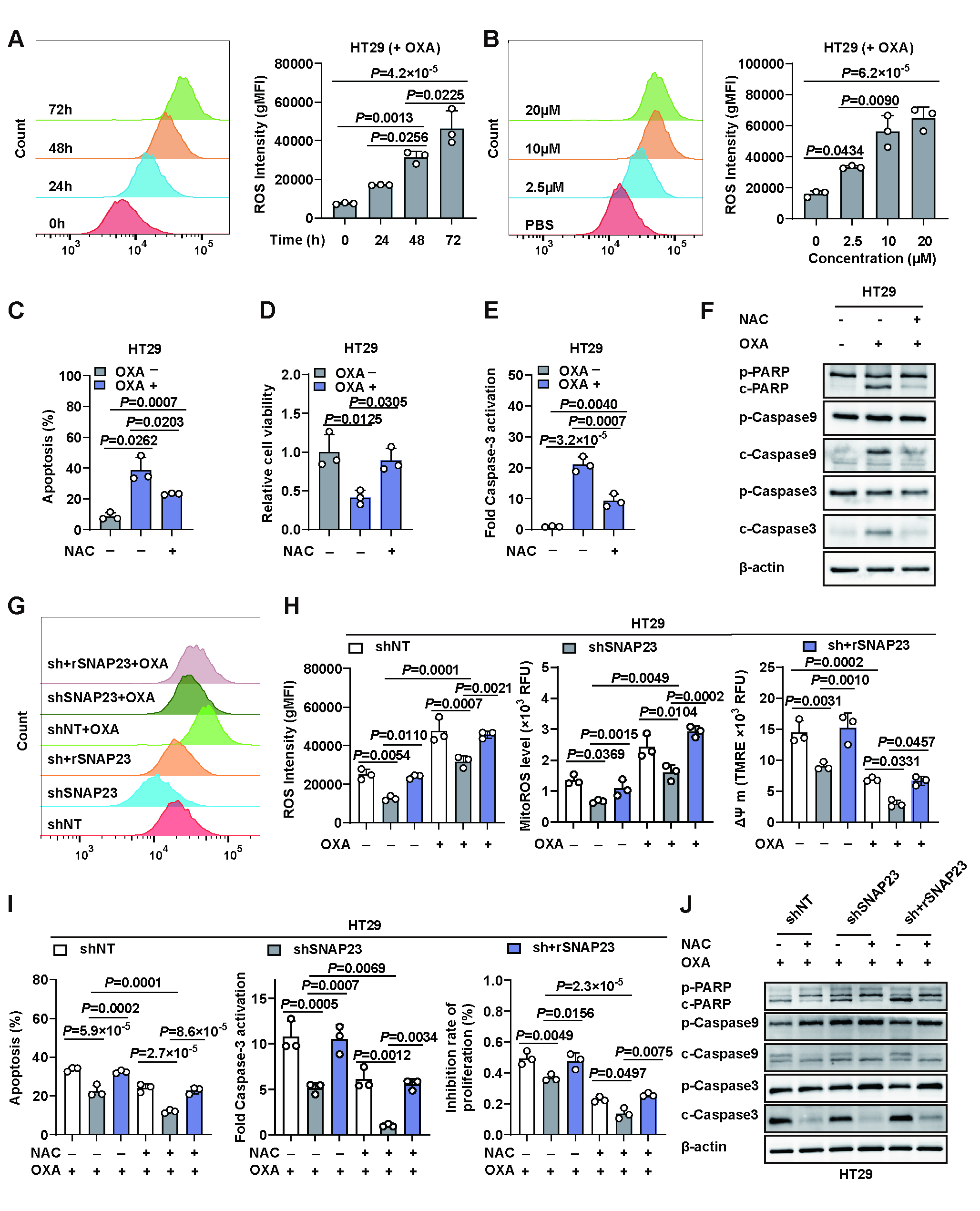

Supplement: Supplementary file 3 — Supplementary Figure 2 [file 41419_2025_8252_MOESM3_ESM.tif]

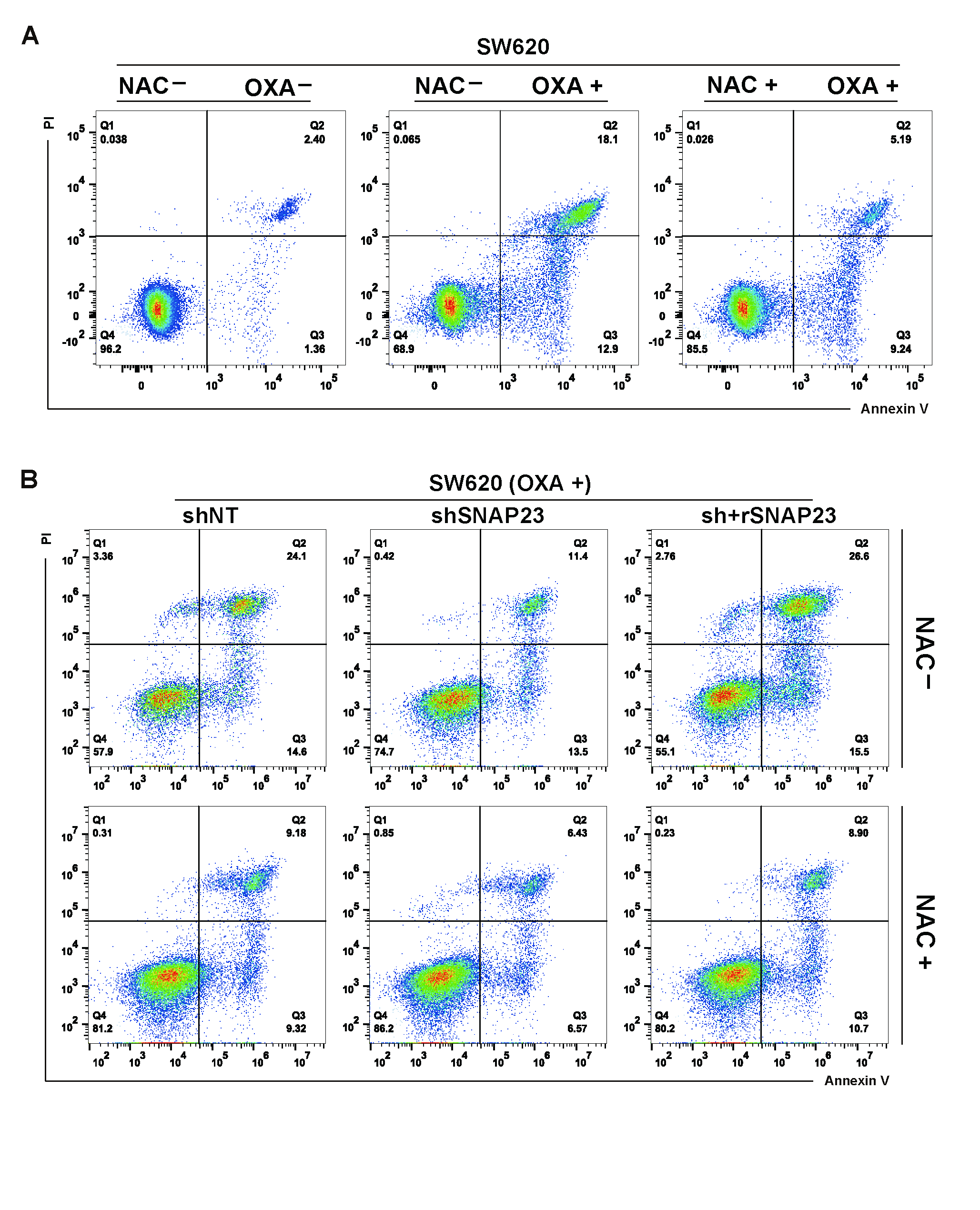

Supplement: Supplementary file 4 — Supplementary Figure 3 [file 41419_2025_8252_MOESM4_ESM.tif]

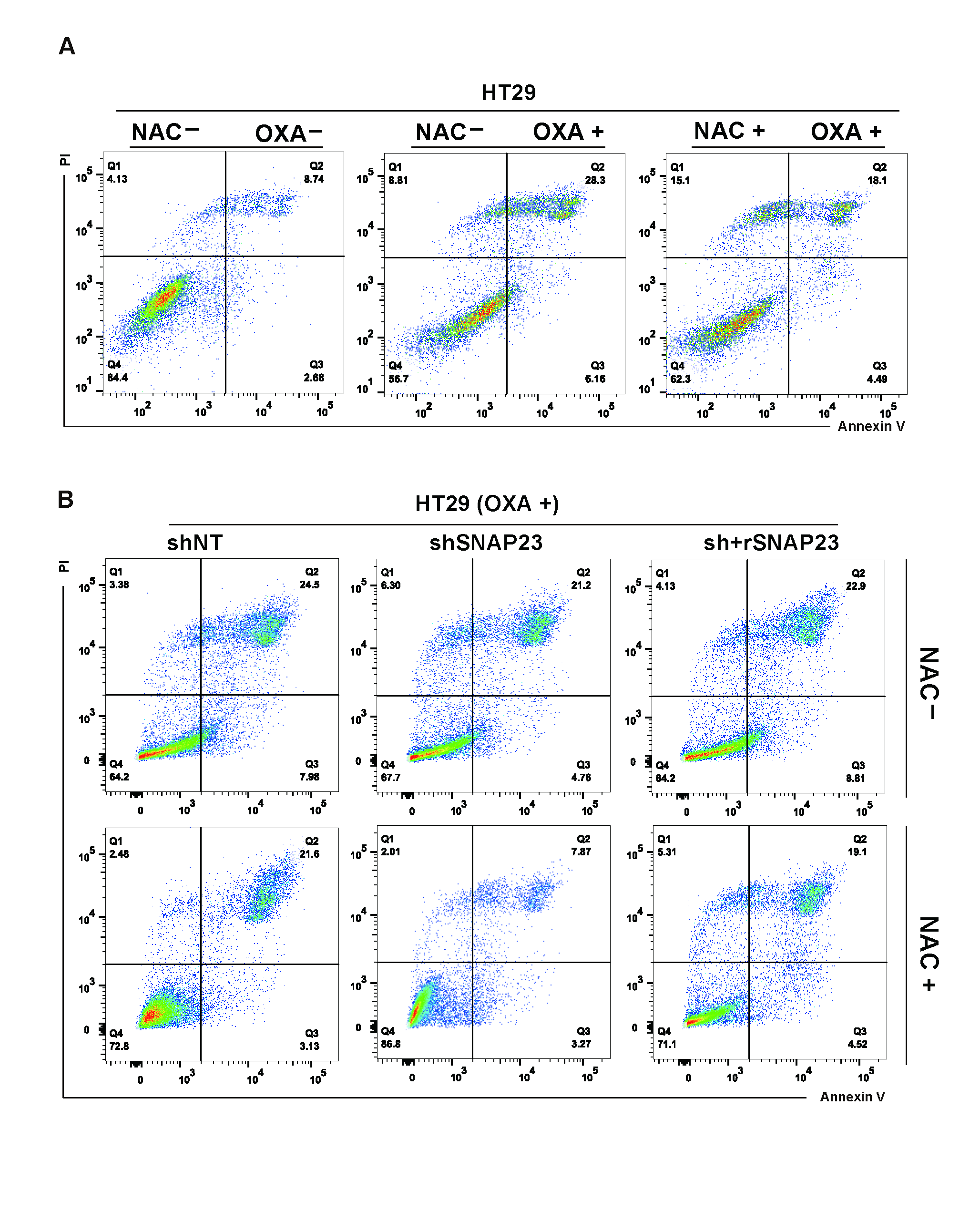

Supplement: Supplementary file 5 — Supplementary Figure 4 [file 41419_2025_8252_MOESM5_ESM.tif]

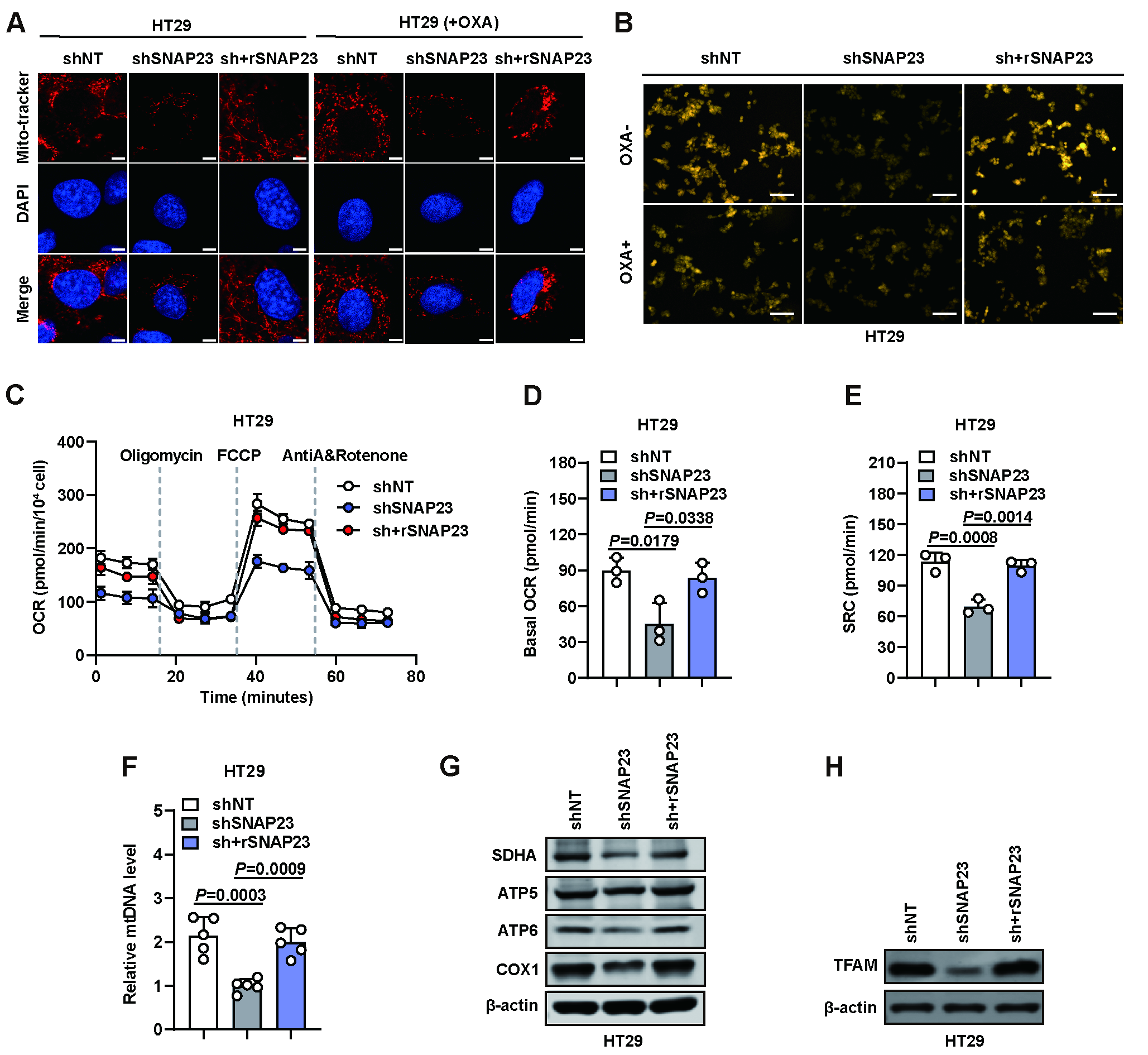

Supplement: Supplementary file 6 — Supplementary Figure 5 [file 41419_2025_8252_MOESM6_ESM.tif]

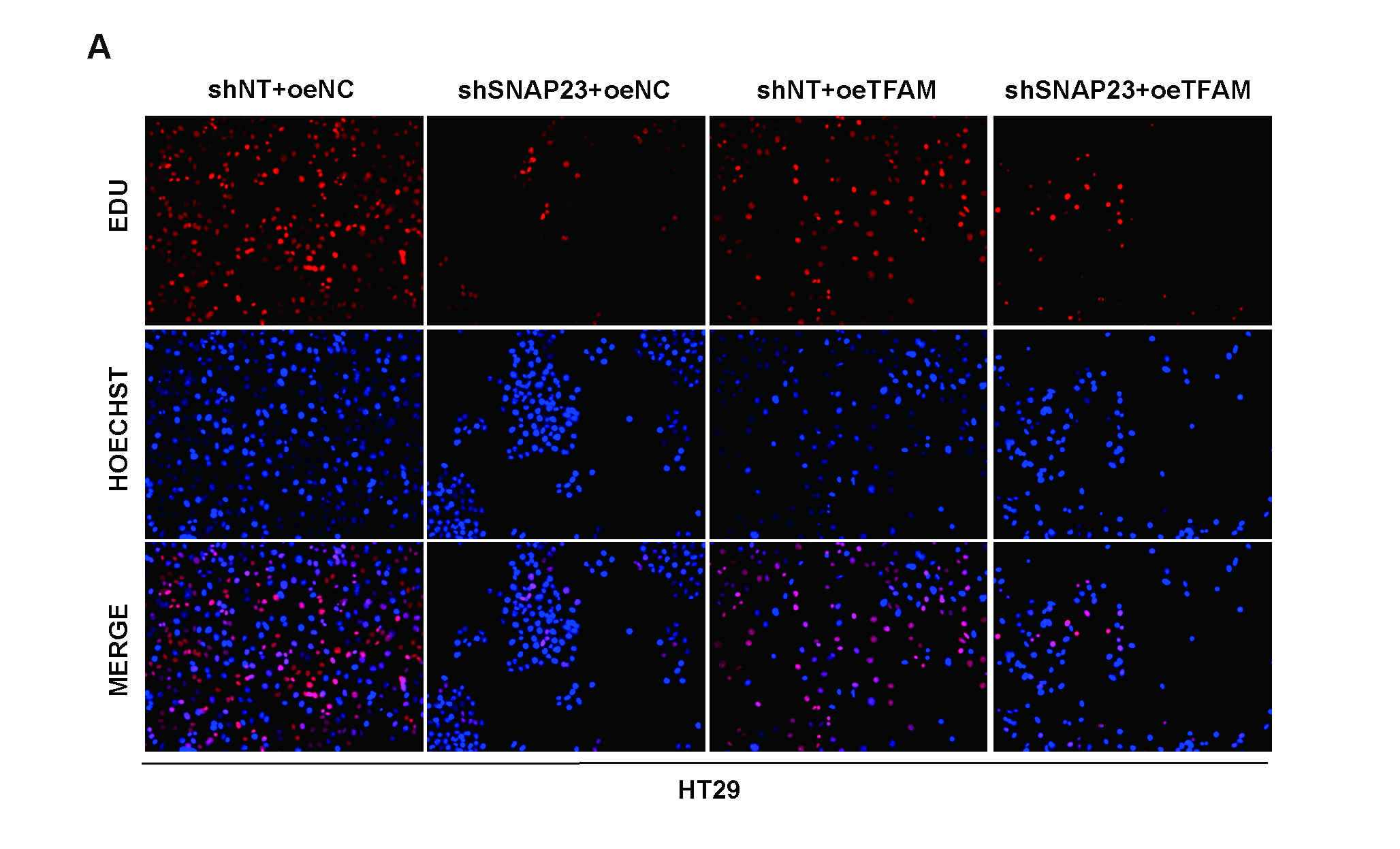

Supplement: Supplementary file 7 — Supplementary Figure 6 [file 41419_2025_8252_MOESM7_ESM.tif]

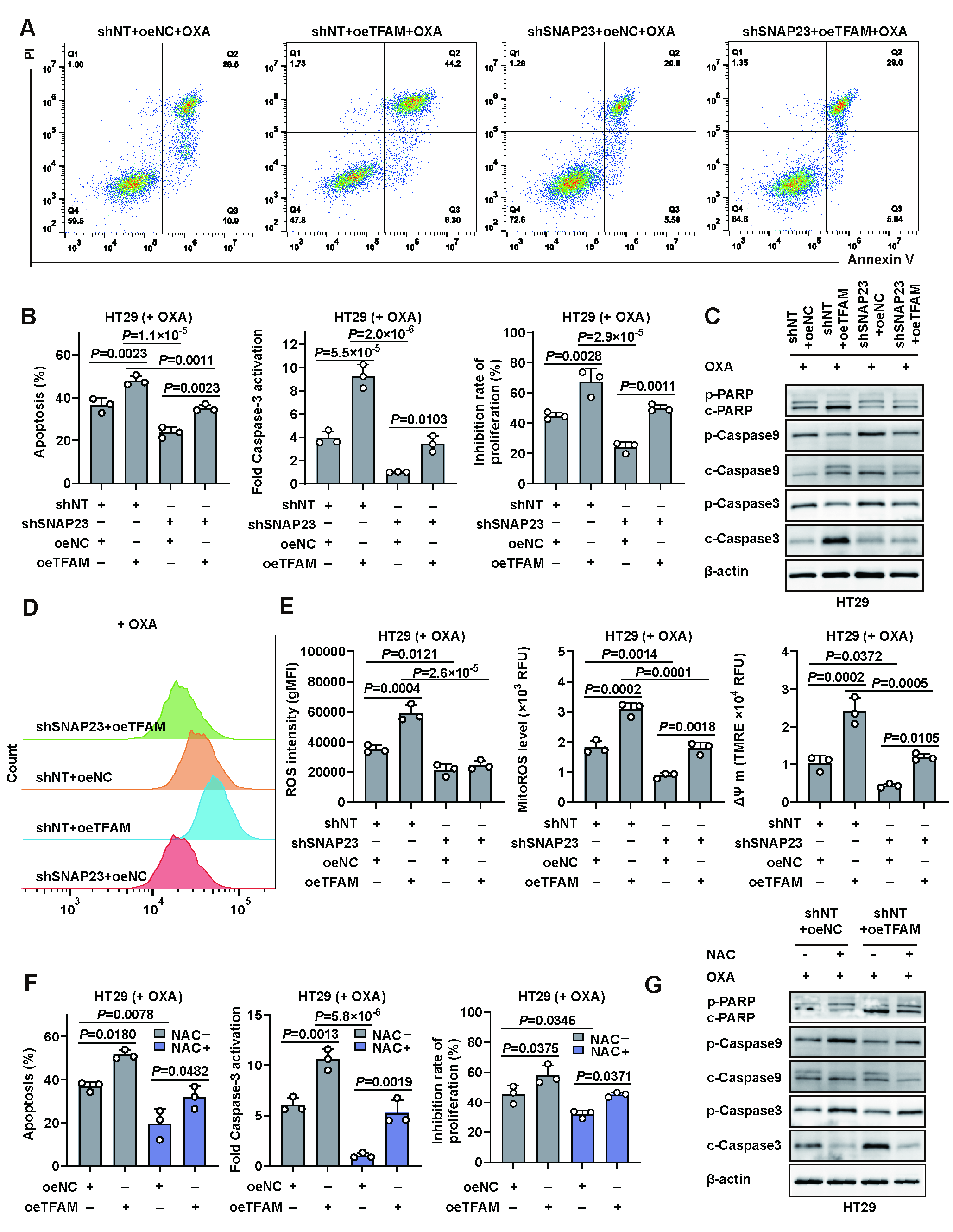

Supplement: Supplementary file 8 — Supplementary Figure 7 [file 41419_2025_8252_MOESM8_ESM.tif]

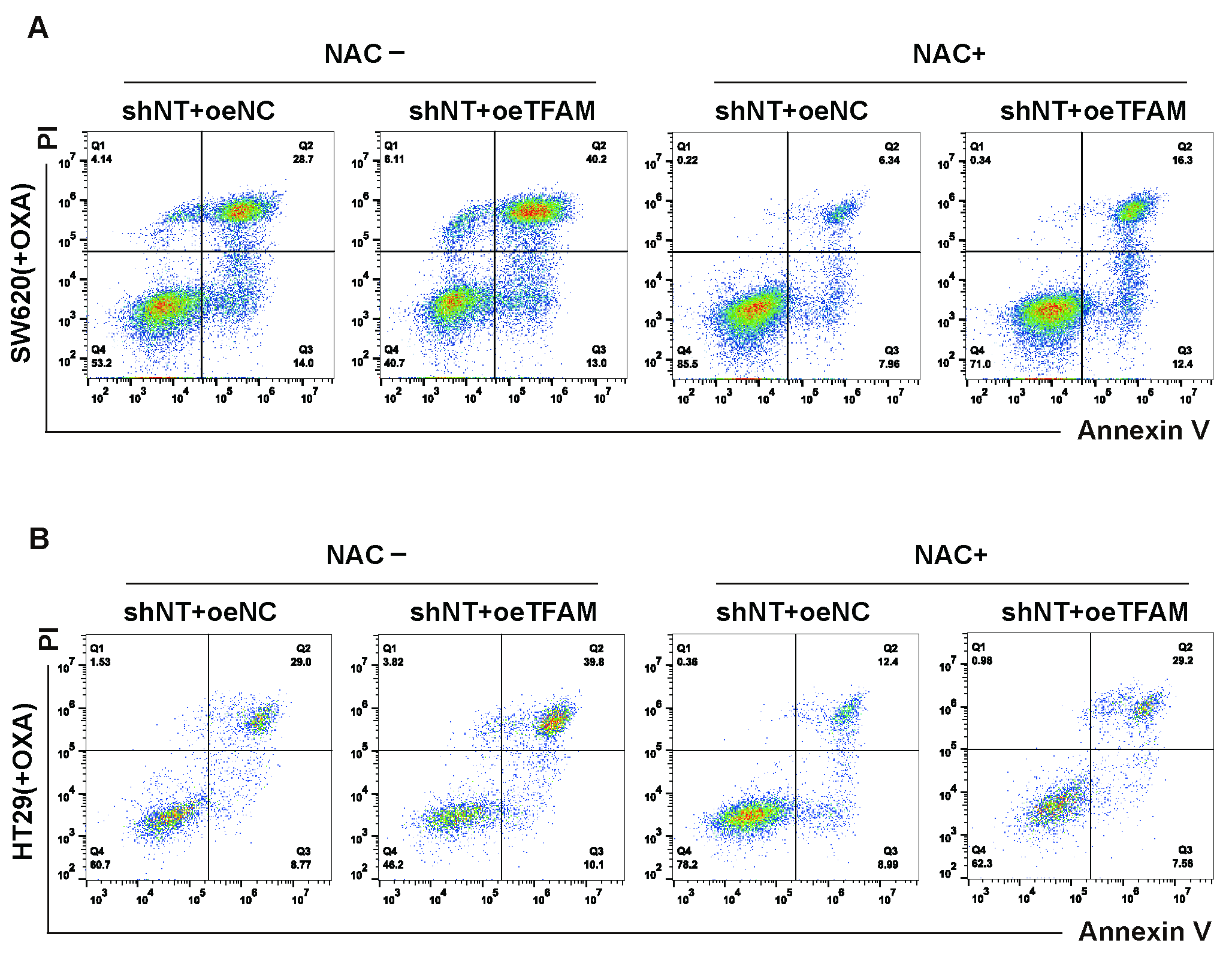

Supplement: Supplementary file 9 — Supplementary Figure 8 [file 41419_2025_8252_MOESM9_ESM.tif]

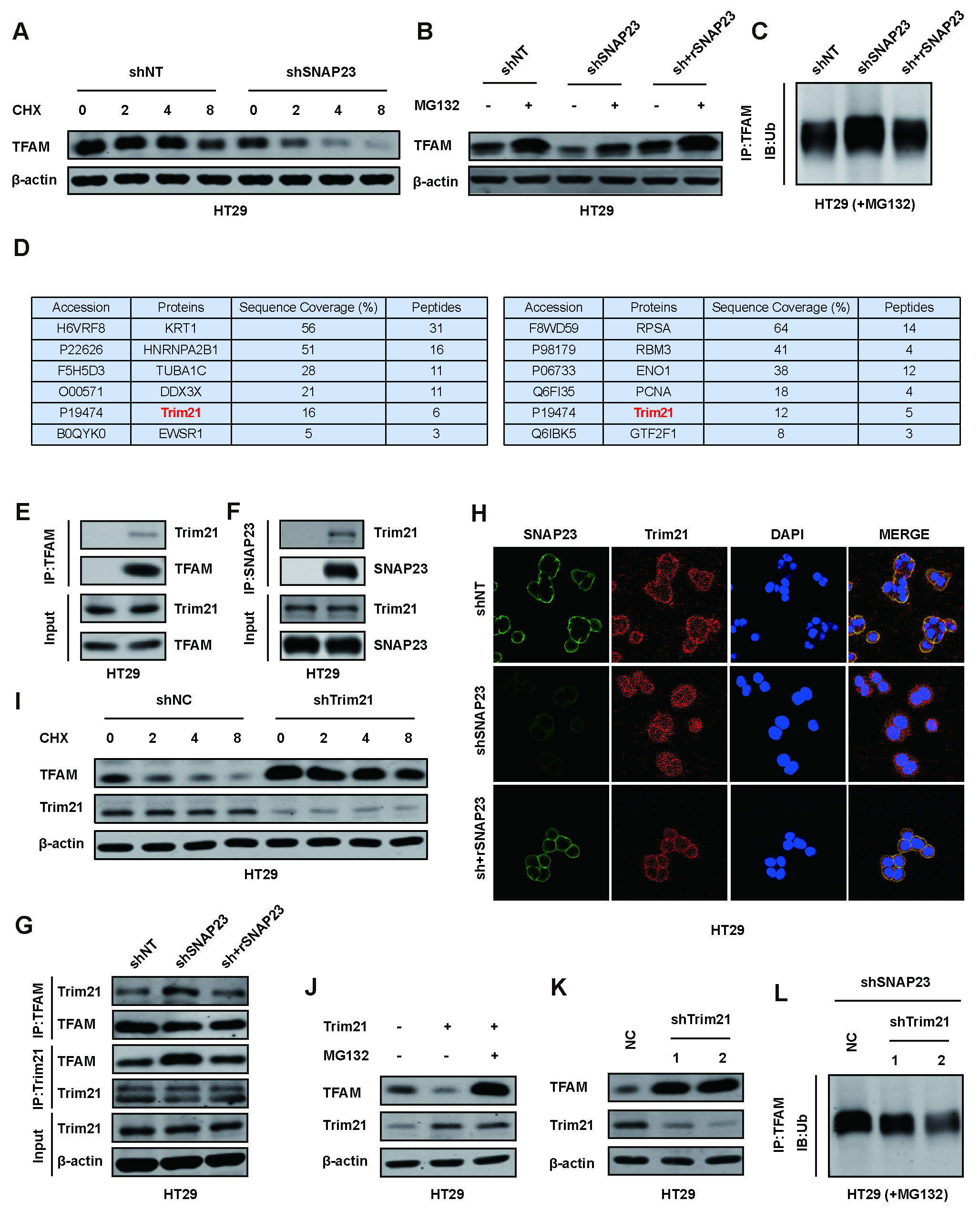

Supplement: Supplementary file 10 — Supplementary Figure 9 [file 41419_2025_8252_MOESM10_ESM.tif]
