# Supplementary material for: SNAP23 deficiency triggers Trim21 mitochondrial translocation to suppress TFAM-mediated oxidative metabolism and drive chemoresistance in colorectal cancer
Source: Cell Death Dis. 2025 Nov 22;17(1):52. doi: 10.1038/s41419-025-08252-1 (PMC12811313; doi:10.1038/s41419-025-08252-1)

Figure 1G

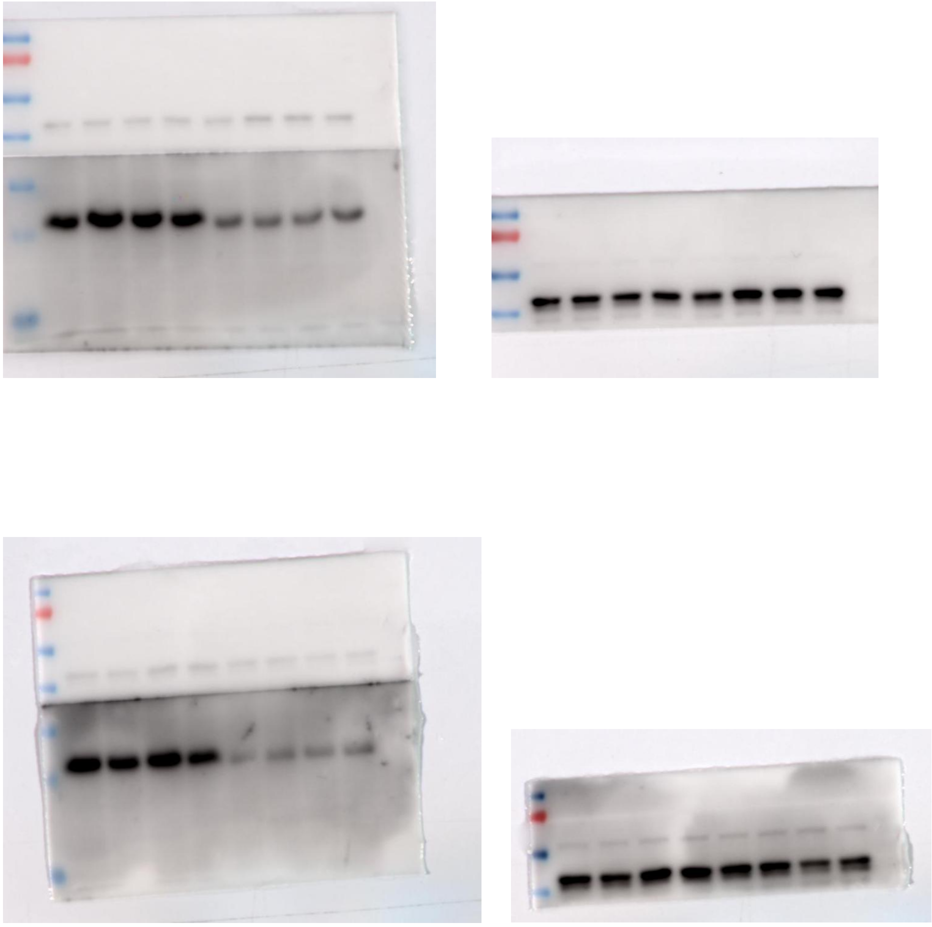

Figure 2A

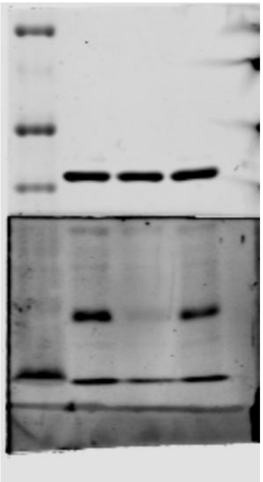

Figure 2D

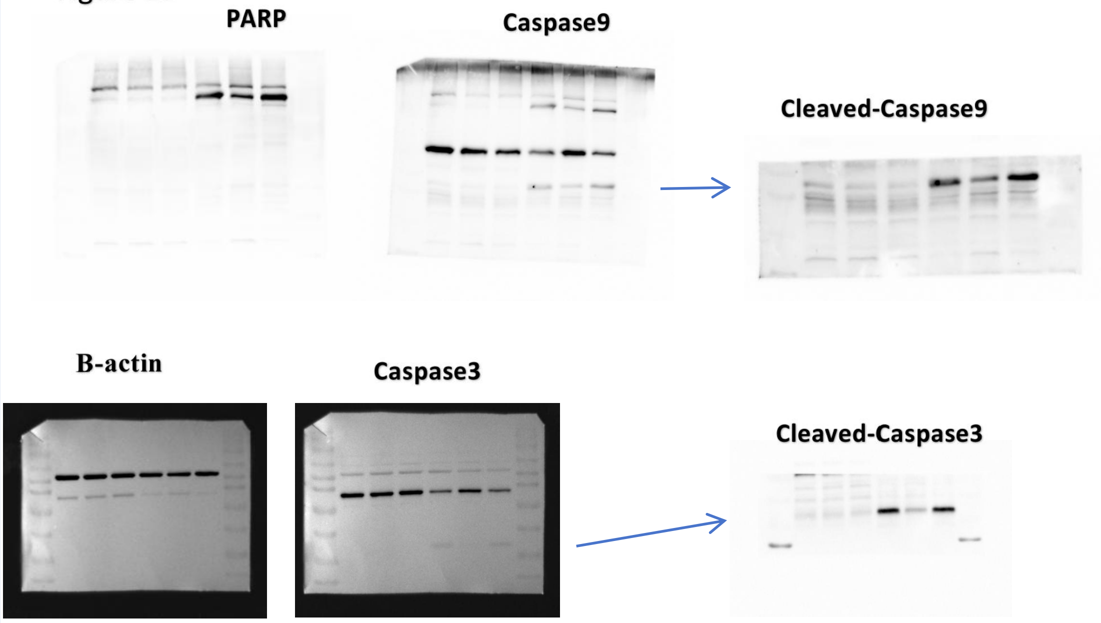

Figure 3F Figure S3F

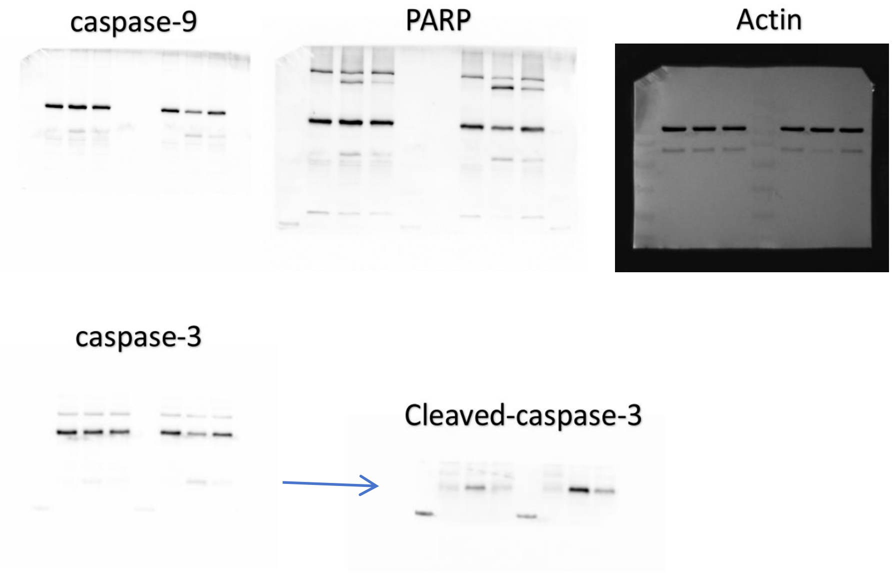

Figure 3K

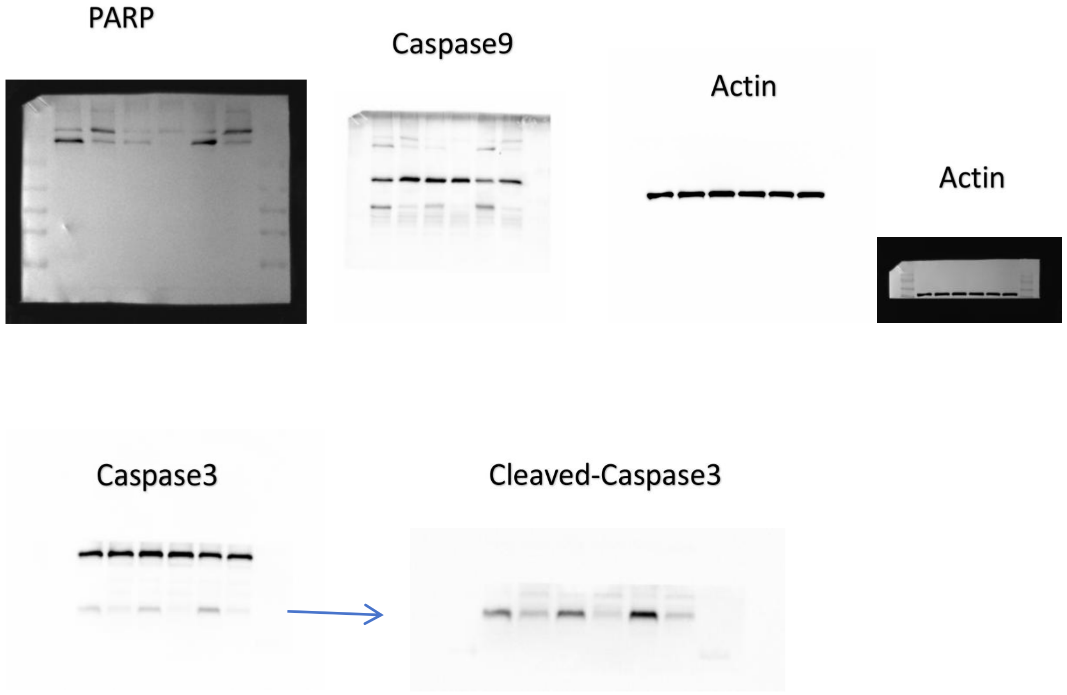

Figure 4H

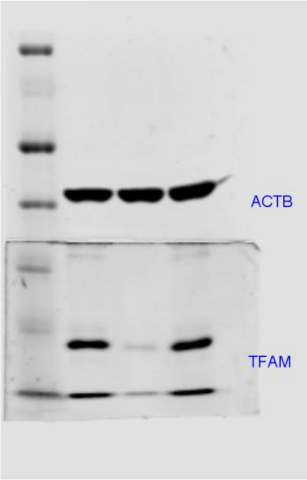

Figure 4G

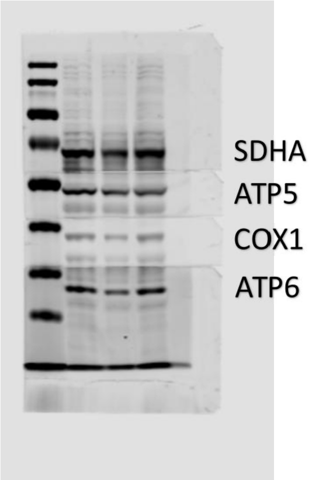

Figure 4G

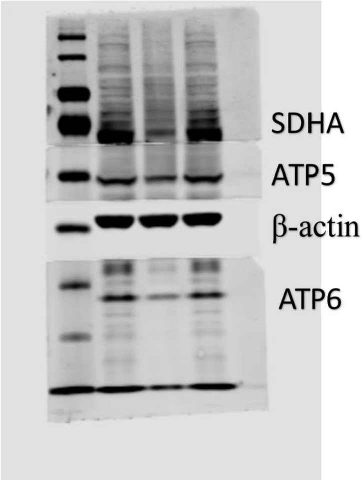

Figure 5D and Figure 5G

Caspase9

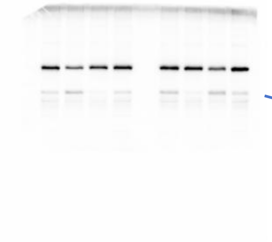

Cleaved-Caspase9

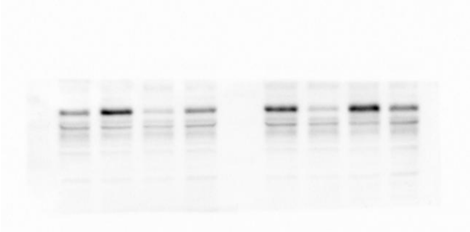

PARP

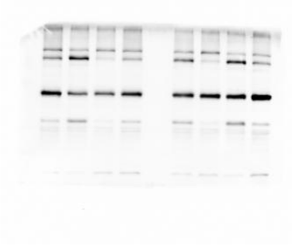

Caspase3

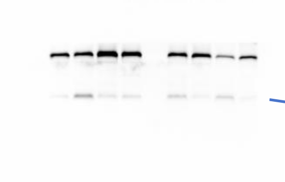

Cleaved-caspase3

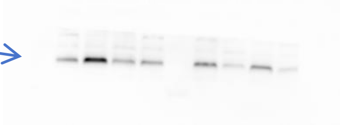

Actin

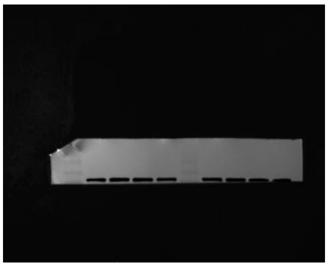

Figure 7A

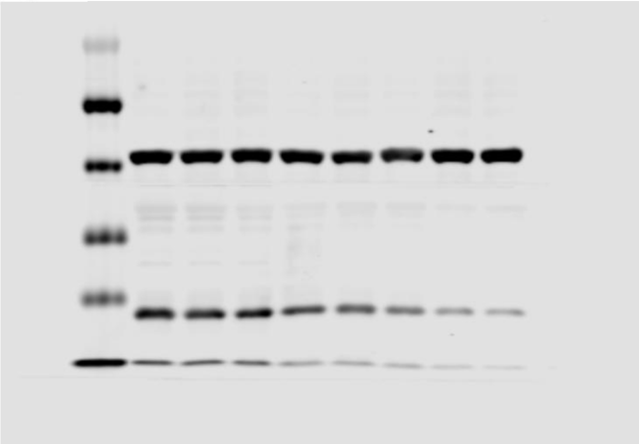

Figure 7B

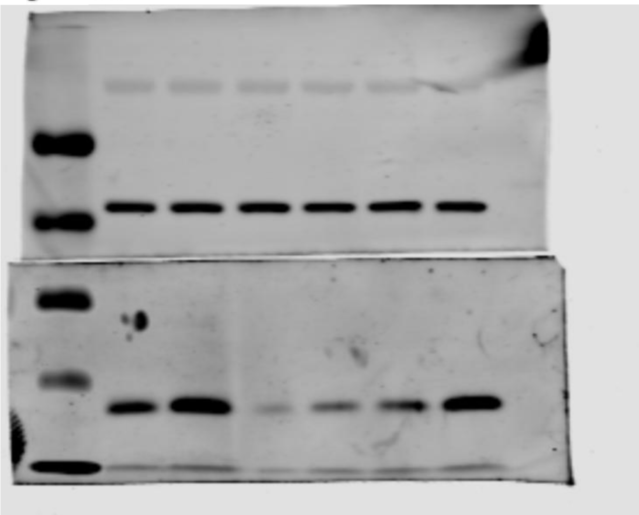

Figure 7C

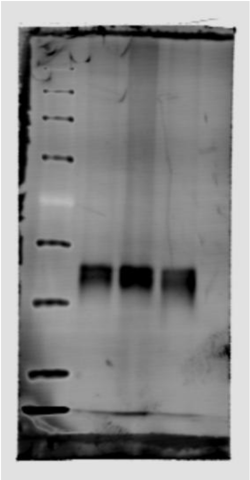

Figure 7E

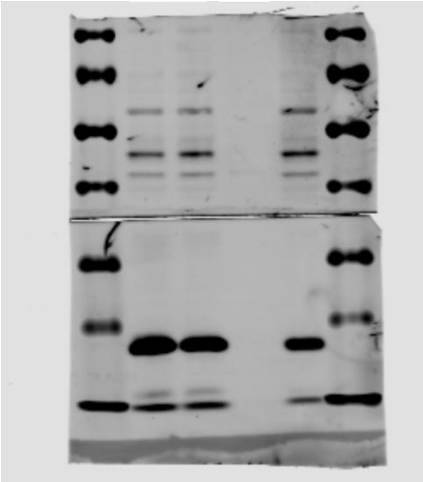

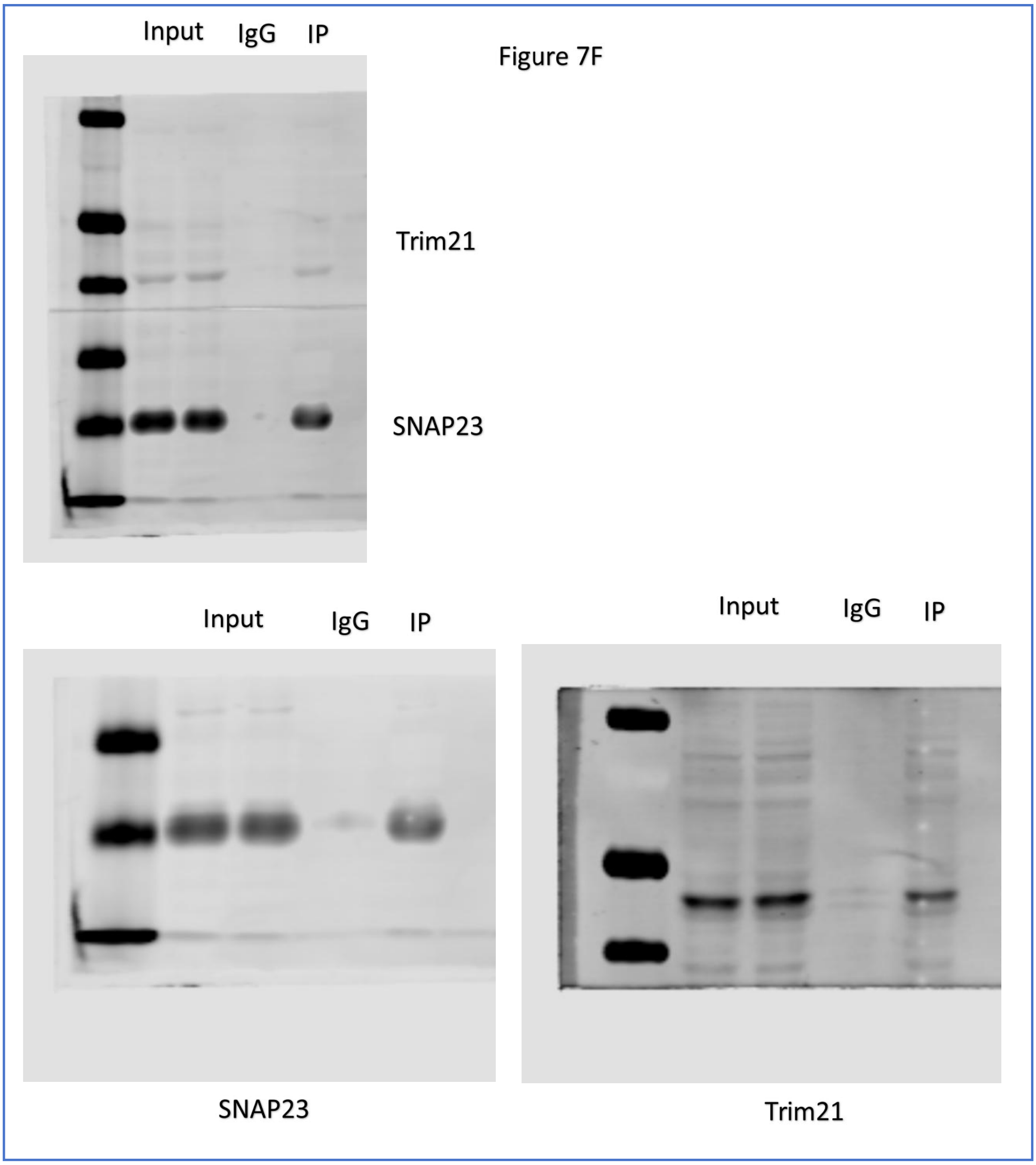

Input
IgG
IP
Figure 7F
Trim21
SNAP23
Input
IgG
IP
Input
IgG
IP
SNAP23
Trim21

Figure 7G

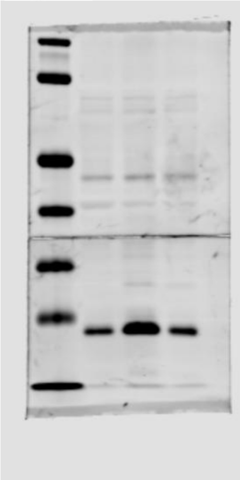

IP:Trim21

Figure 7G Trim21

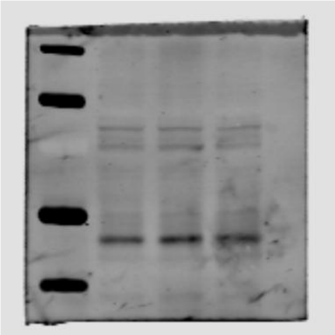

Figure 7G TFAM

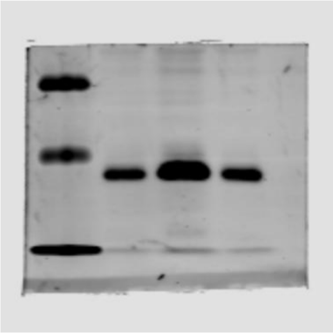

Figure 7G

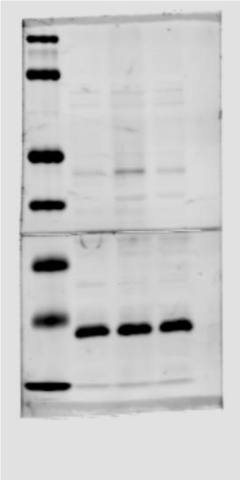

IP:TFAM

Figure 7G Trim21

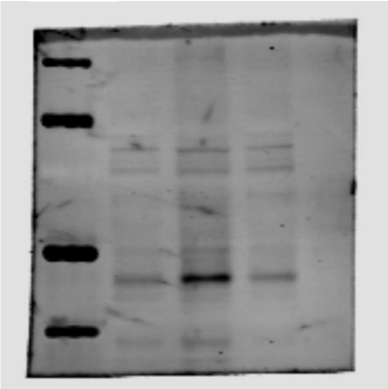

Figure 7G TFAM

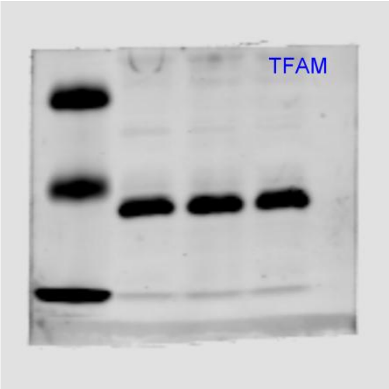

Figure 7G Input

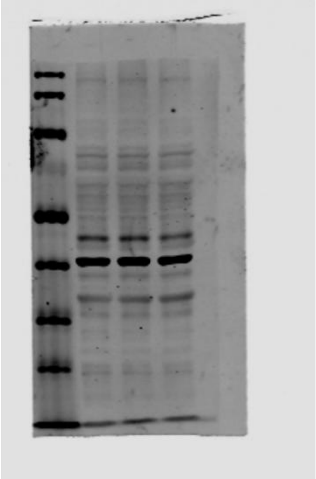

Figure 7H

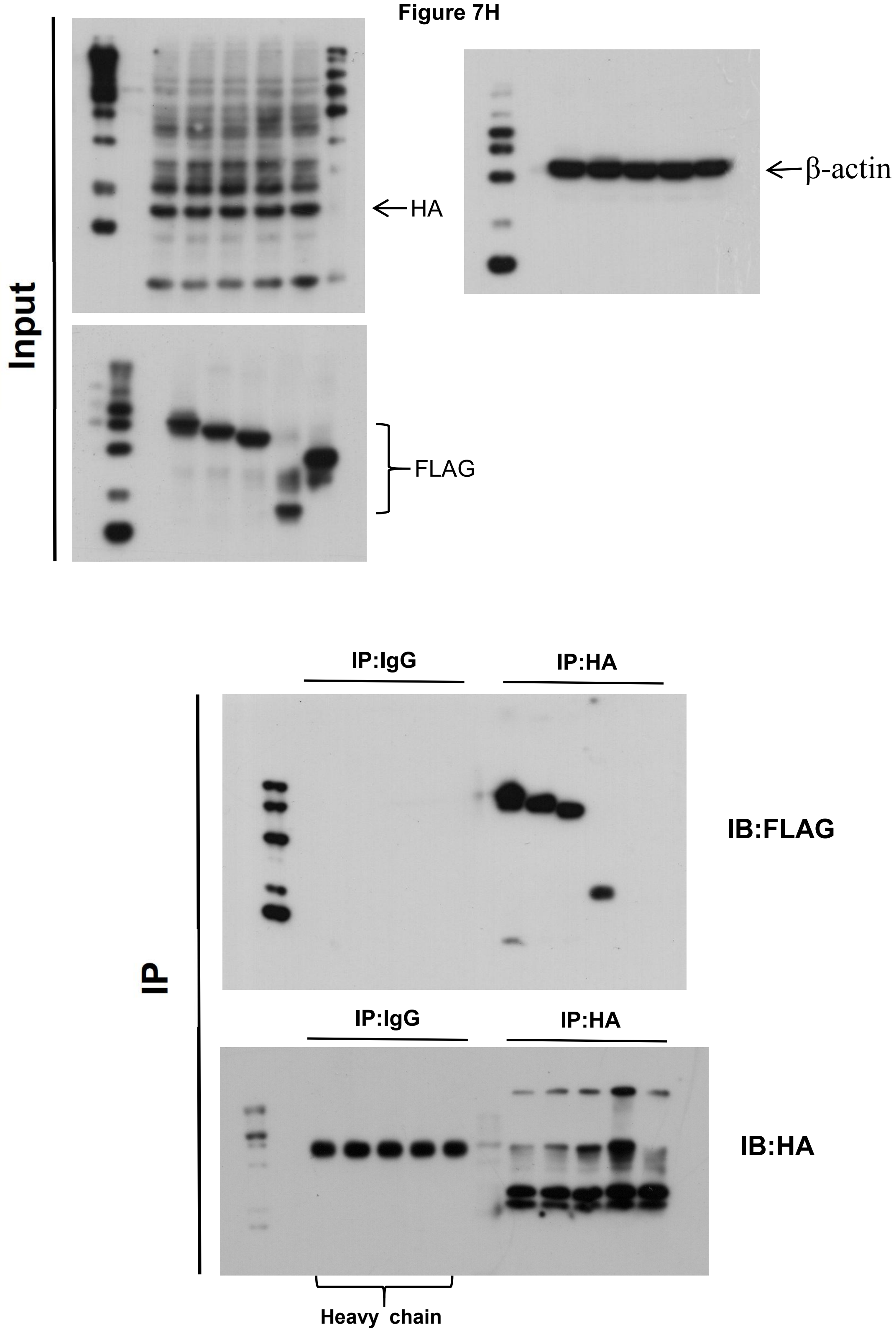
Input
HA
β-actin
FLAG
IP
IP:IgG
IP:HA
IB:FLAG
IP:IgG
IP:HA
IB:HA
Heavy chain

Figure 7I

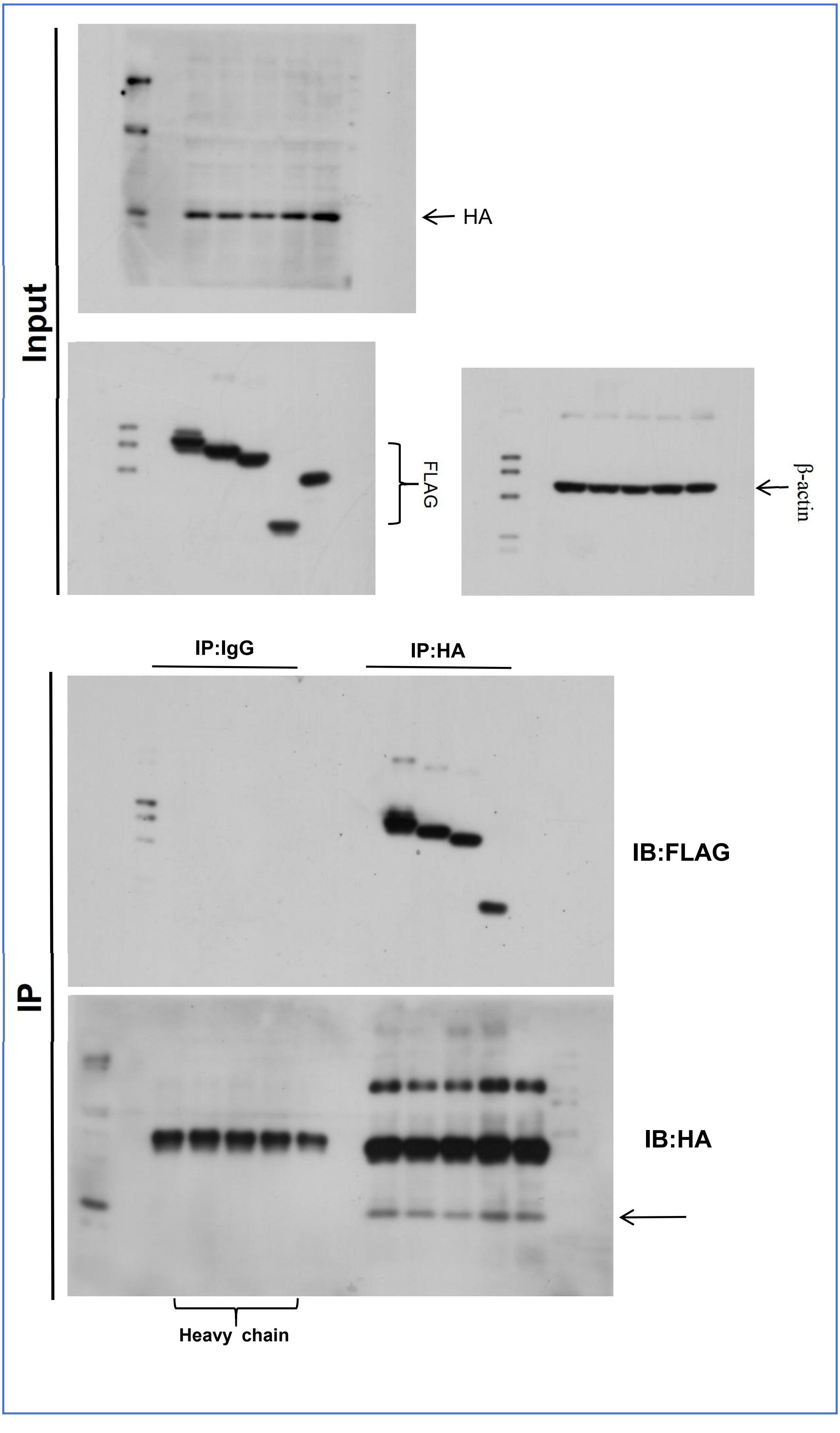

Figure 7J

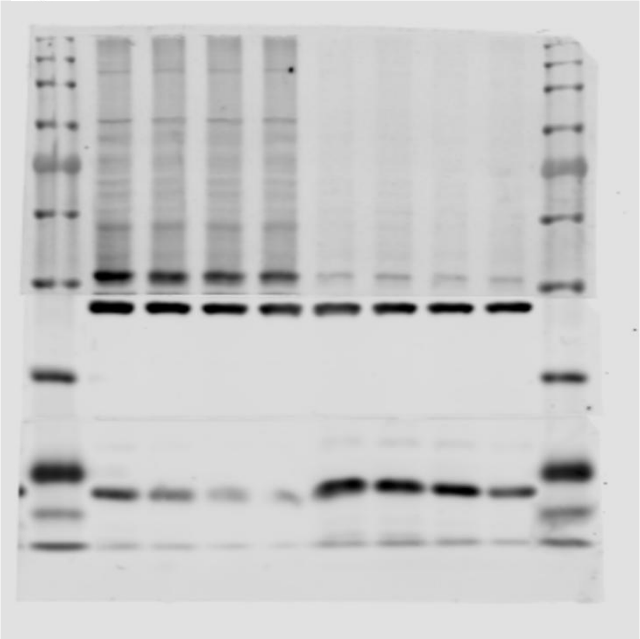

Figure 7K

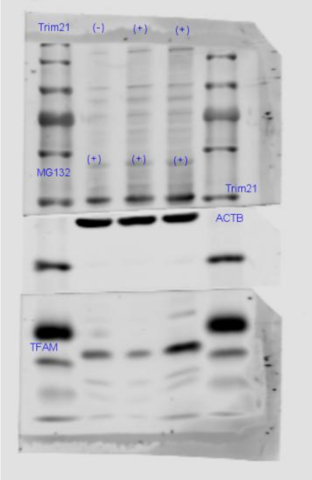

Figure 7L

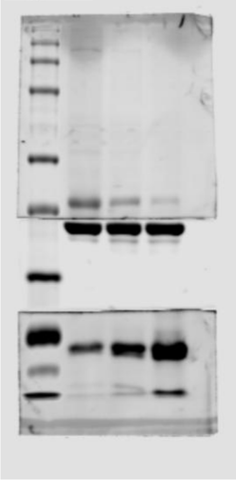

Figure 7M

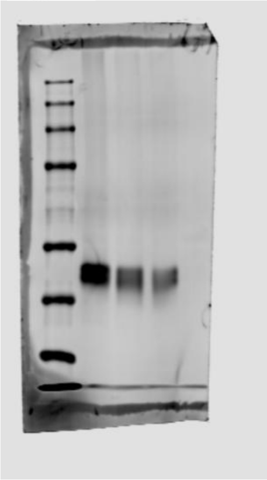

Figure 7E

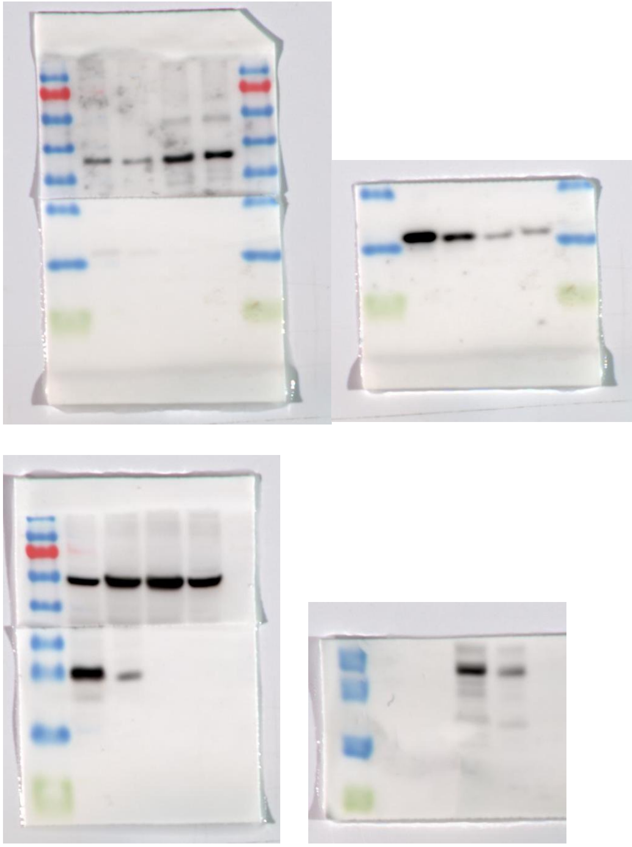

Figure 7F

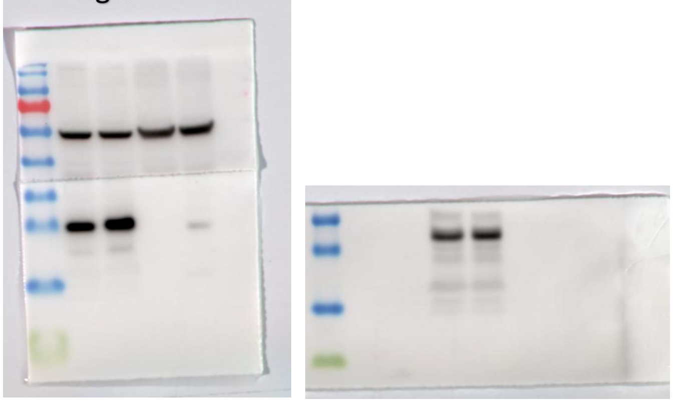

Figure 7G

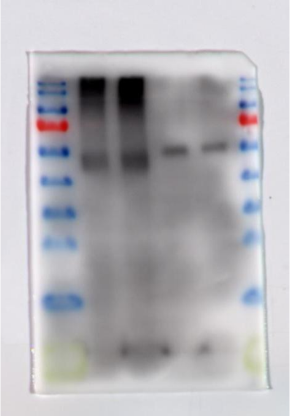

Figure S1A

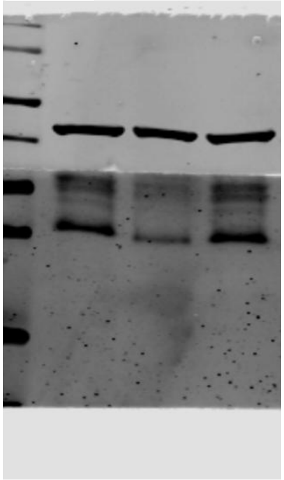

Figure S1D

PARP

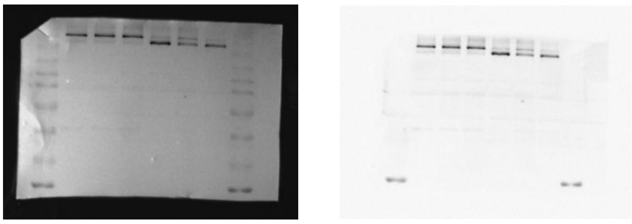

Caspase9

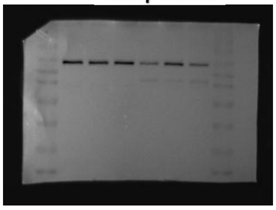

Caspase9

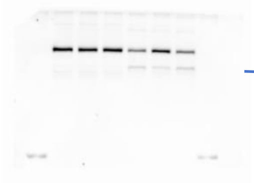

Cleaved-Caspase9

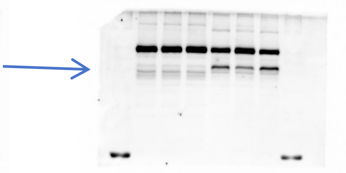

Actin

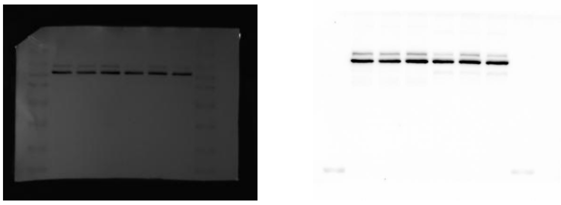

Caspase3

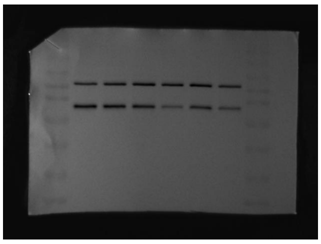

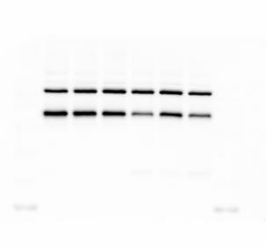

Cleaved-Caspase3

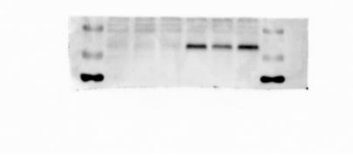

Figure S2J

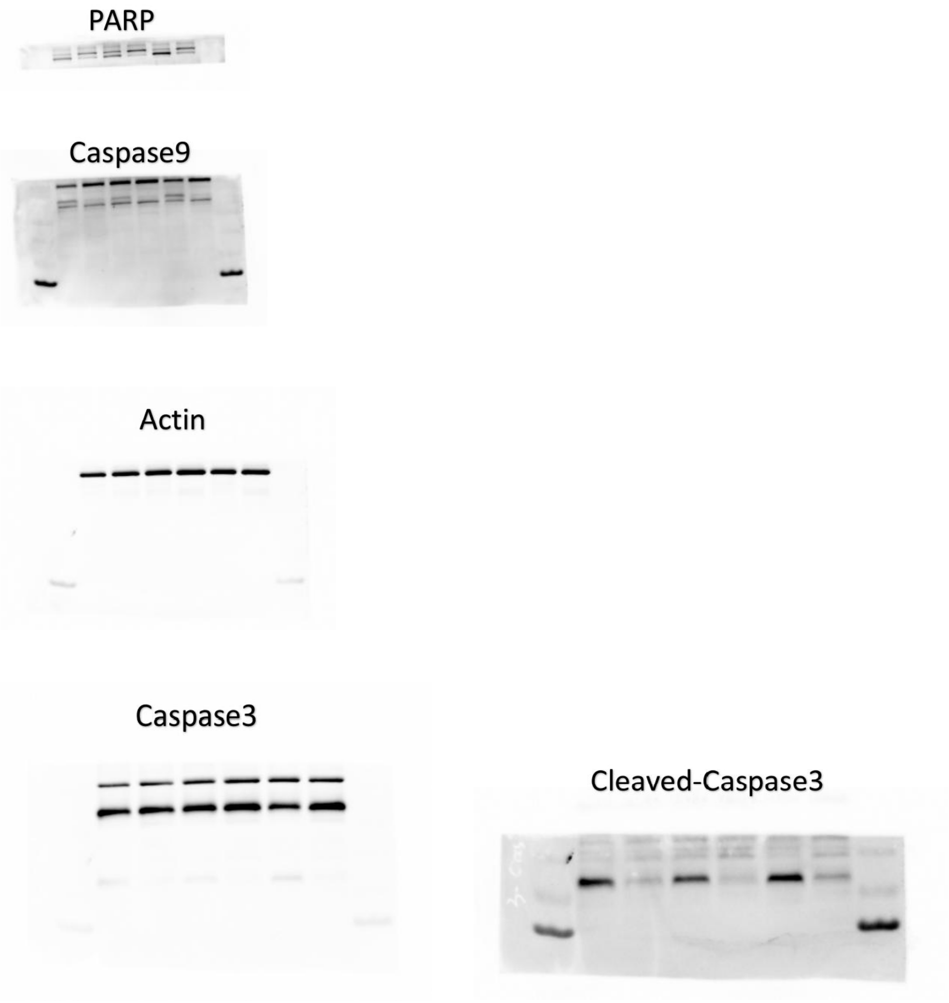

Figure S5G

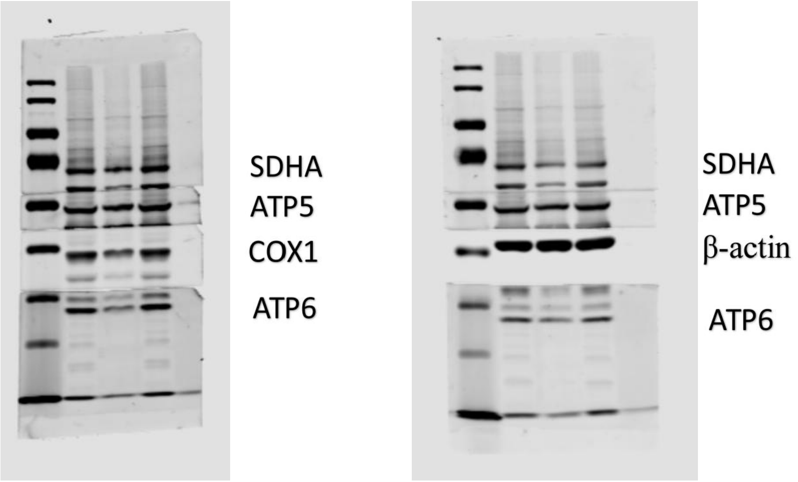

Figure S5H

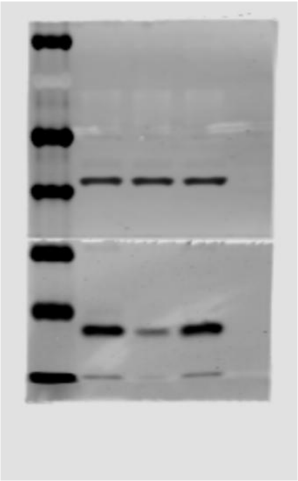

PARP

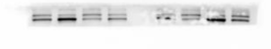

Figure S7C and Figure S7G

Caspase9

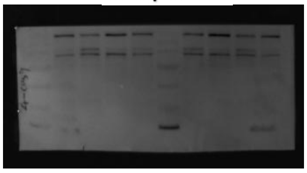

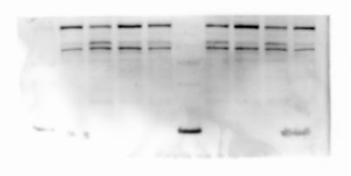

Actin

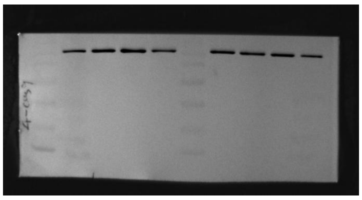

Caspase3

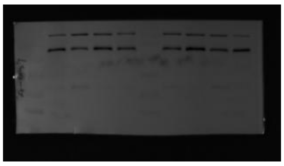

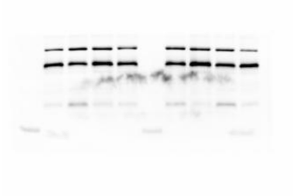

Cleaved-Caspase3

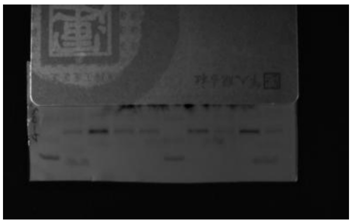

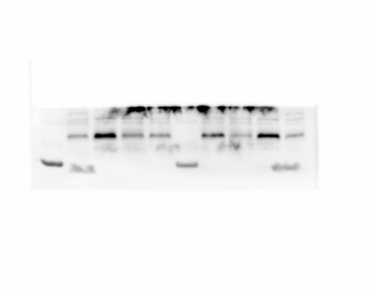

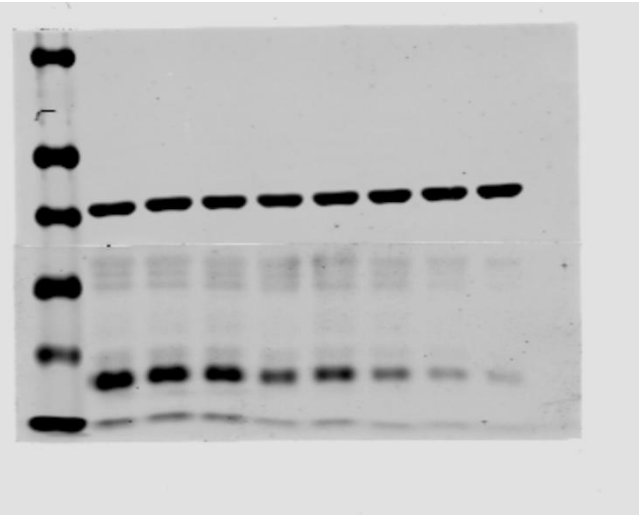

Figure S9A

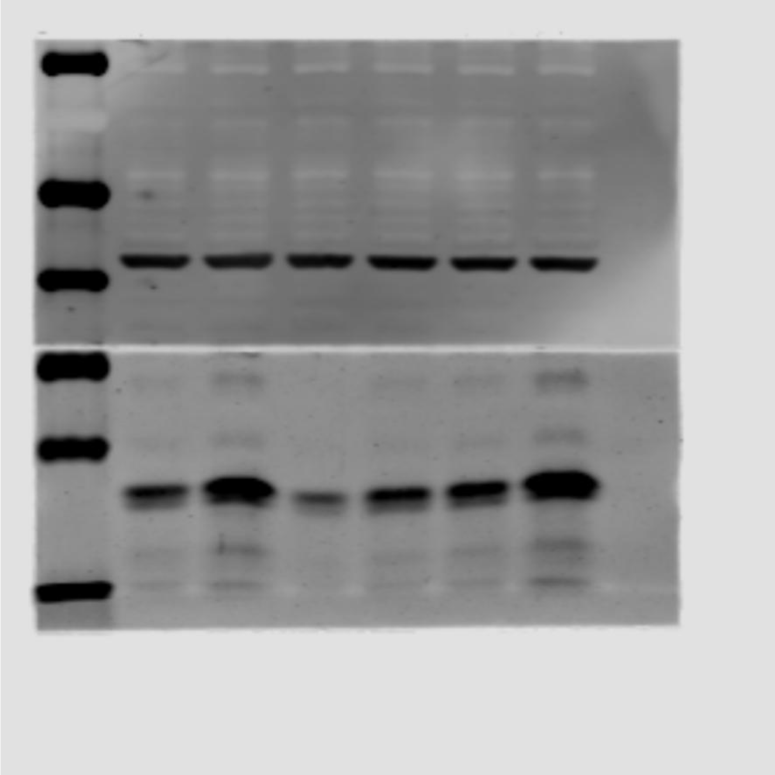

Figure S9B

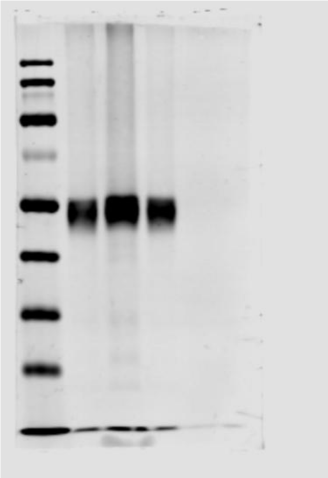

Figure S9C

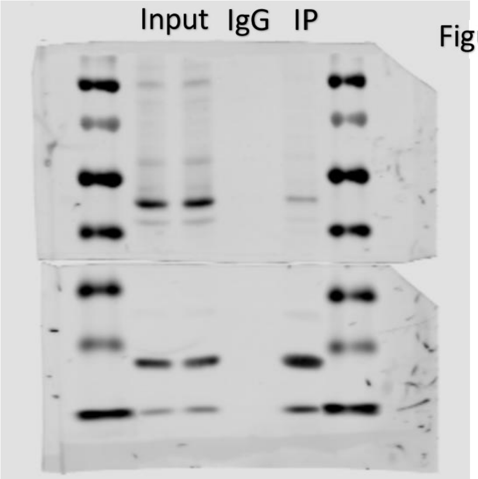

Figure S9E

Figure S9F

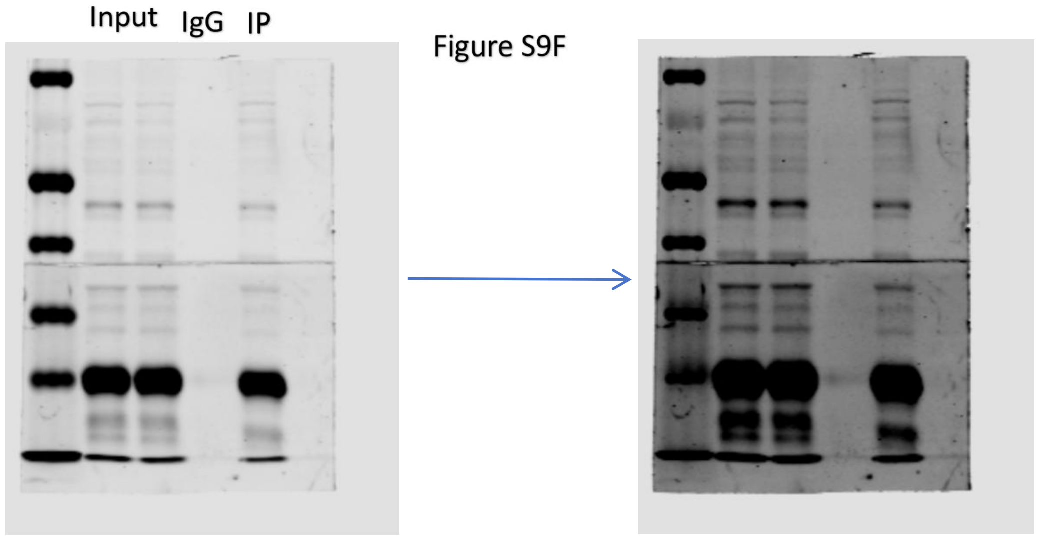

Figure S9G

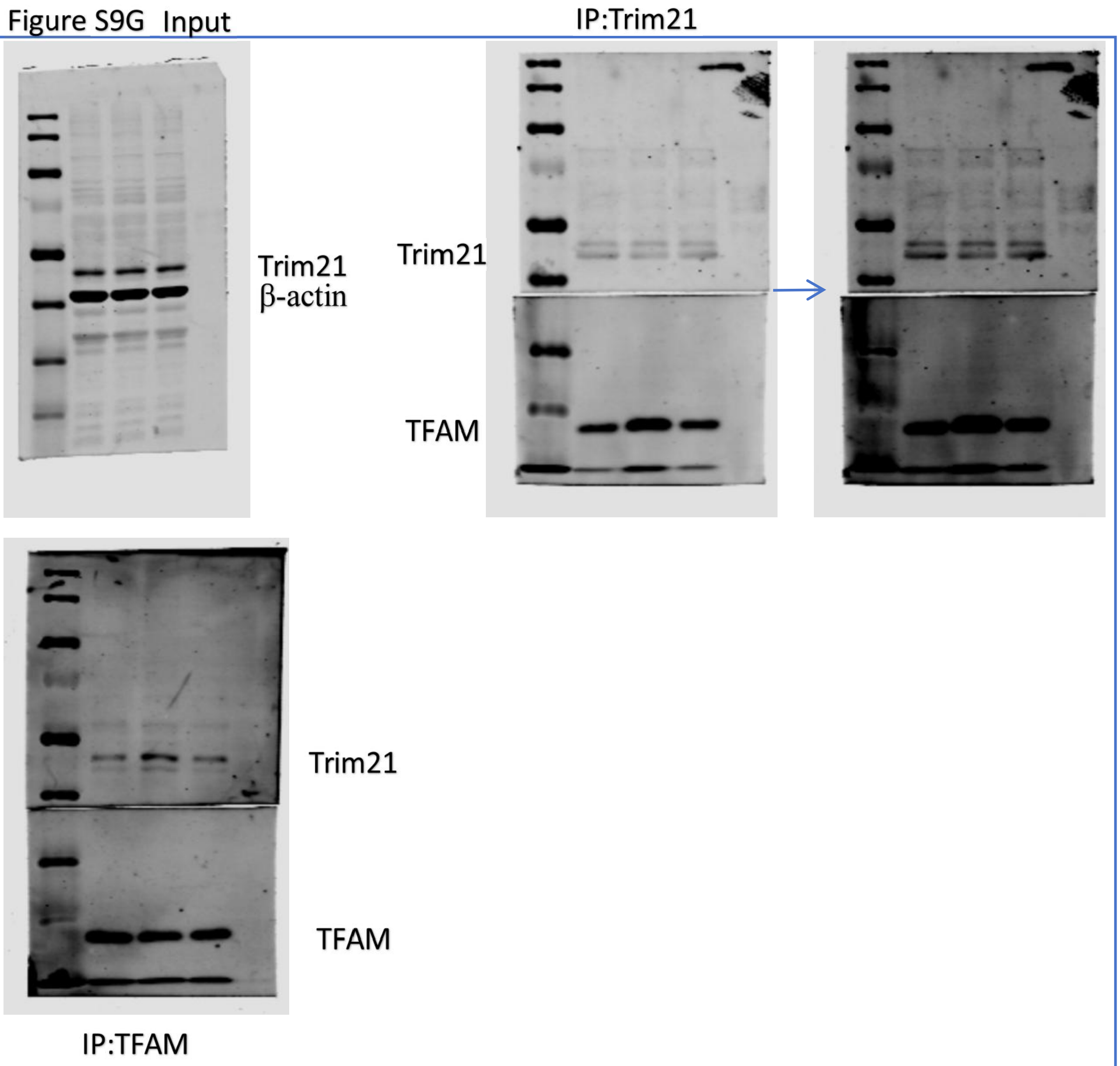

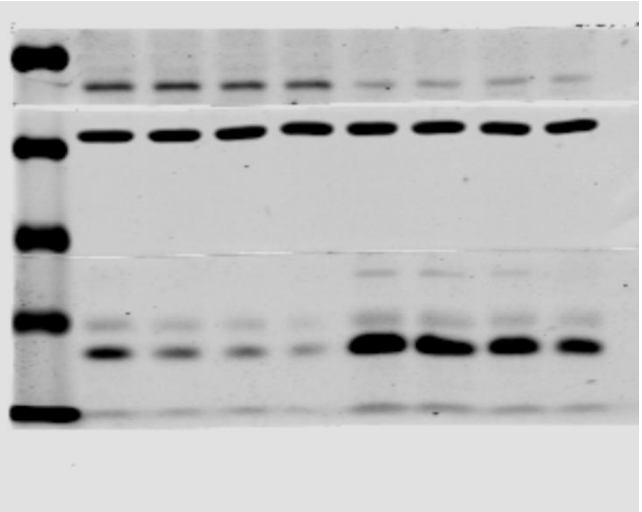

Figure S9I

Figure S9J

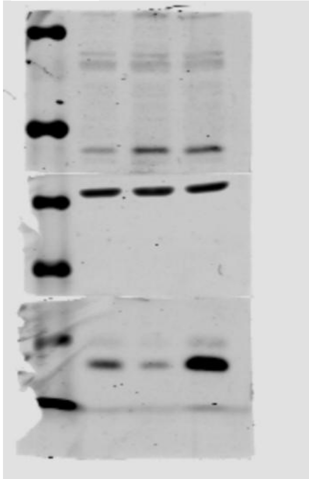

Figure S9K

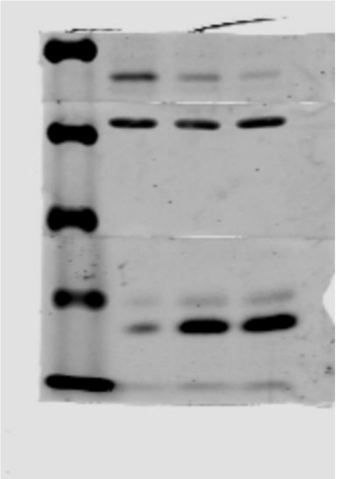

Figure S9L

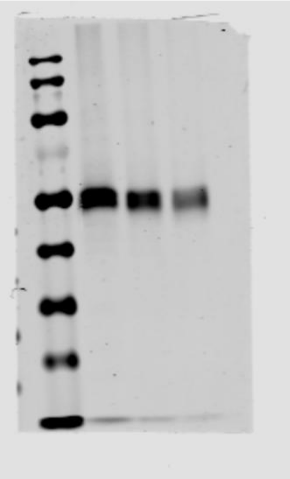

Supplement: Supplementary file 11 — Original Western Blots [file 41419_2025_8252_MOESM11_ESM.pdf]
